# A Geometric-Based LSGDM Method for Tourism Project Decision Optimization with Trust–Distrust Relationships

**DOI:** 10.3390/e24050588

**Published:** 2022-04-22

**Authors:** Lei Zhou, Xinshang You, Shuo Zhao, Zengtai You

**Affiliations:** 1 School of Economics and Management, Hebei University of Science and Technology, Shijiazhuang 051432, China; zhoulei19@hebust.edu.cn (L.Z.); zhaoshuo1104@stu.hebust.edu.cn (S.Z.); 2 Information Technology and Cultural Management Institute, Hebei Institute of Communications, Shijiazhuang 051432, China

**Keywords:** trust–distrust relationship, large-scale group decision-making, interval-valued intuitionistic fuzzy number, tourism project decision optimization

## Abstract

In this paper, we discuss the decision optimization of tourism projects in Hebei Province, China. To improve the process of analyzing tourism projects, we introduce a model that includes multiple decision makers as subjects based on a standard four-dimensional evaluation system. In order to improve the effectiveness of decision-making results, we will increase the number of decision makers to 40. A novel large-scale group decision-making (LSGDM) algorithm that incorporates the trust–distrust asymmetric relationships between decision makers is proposed. This model contains three main innovations: firstly, in the evaluation of decision makers’ social network relations, the trust–distrust value is introduced as a new carrier, and a weighted directed network and data integration operator are constructed based on the evaluation between decision makers; secondly, an extended Girvan-Newman (GN) algorithm is constructed to cluster the decision makers from this weighted network; thirdly, the interval-valued intuitionistic fuzzy number (IVIFN) is used to evaluate the alternatives, studying the IVIFN’s geometric significance by placing in a rectangular coordinate system. Finally, a new LSGDM model is proposed. Using the development of a cultural tourism project in a township as an example, the effectiveness of the proposed model is illustrated. By comparing the results of our method to those of a LSGDM algorithm that does not incorporate trust relationships, we assess the performance of the new model.

## 1. Introduction

How to develop sustainably is a global topic. Environmental advocates from national governments and civil liberties organizations around the world are increasingly attaching importance to sustainable development and constantly emphasizing the importance of environmental protection. China has taken environmental protection as a basic state policy for national development and promulgated laws on environmental protection at the national level. Chinese President Xi Jinping pointed out that China will continue to promote sustainable development and fully implement the UN 2030 Agenda for Sustainable Development. China will strengthen ecological progress and accelerate the optimization of industrial structure. The capital of China, Beijing, is surrounded by Hebei Province, where the construction of ecological civilization system is of greater significance. Hebei Province has almost all kinds of tourism natural landscapes mentioned in the case study section. In addition, the natural environment of Hebei Province is not good, gradually developing the tourism industry for improving. “Improving the construction of the ecological environment is not only the requirement for the development of HeBei Province in China but also the need of serving the overall situation of the country.” The provincial Party Committee and the provincial government of Hebei pointed out that Hebei must follow the road of green development. Reasonable development of cultural tourism projects can meet the needs of the people in pursuit of a better life and can also improve social and ecological civilization. The overall pattern of tourist influence in Hebei Province shows an increasing trend, but there is still a large space for improvement [1].

Since 2021, because the continued loose fiscal and monetary policies of major economies and the acceleration of global vaccine production and vaccination, global economic activity has been further active and shown a clear recovery trend. In 2021, the total number of global tourists (including domestic and international tourists) reached 6.60 billion. The total global tourism revenue in 2021 (including domestic and international tourism revenue) will reach USD 3.3 trillion, an increase of USD 0.20 trillion compared with 2020. The year-on-year growth of 6.45% was 55.9% in 2019 before the epidemic. Global tourism recovered in 2021, but the pace is difficult, and the foundation is not yet solid (Source: World Tourism Cities Federation, Zhiyan Consulting, 30 March 2022). The immediate effect of the war was a rise in global commodity prices. In the first few days of the Russia-Ukraine war, gold, oil, etc., rose around 5−10%. Other commodity prices, although they will also have an impact, should generally fluctuate within the above range. Then there is the impact on the stock market. The American and European stock markets began to fall sharply but rebounded sharply on the second day after the fall, and then fluctuated somewhat, not exceeding 3% overall. The same is true for Chinese stocks, but the renminbi continues to appreciate. The Russian central bank immediately raised its benchmark interest rate from 9.5% to 20%. Generally speaking, the impact of the war on the international financial market fluctuates between 3–10% in the short term, and it is estimated that it is difficult to expand the fluctuation range in the long term. This is mainly due to the relatively small economies of Russia and Ukraine, which have limited lasting influence. This is a sudden short-term factor that affects global economic trends and will return to normal in the long run. (Source: Hainan Institute of Green Finance, China). COVID-19 still has had a great influence on the tourism industry. However, as noted by the general secretary, we should judge China’s economy on its long-term development, as well as the cultural and tourism development of our province. This paper will examine cultural tourism from the perspective of ecologically sustainable development, and the scope of this study mainly lies in the areas inhabited by human beings. 

### 1.1. Research on the Construction of Ecological Civilization

A tweet from the National Aeronautics and Space Administration (NASA) on 12 February 2019, stated that the earth is currently greener than it was 20 years ago. The increase in the amount of vegetation in China accounts for at least 25% of the global increase in vegetation over the past 17 years, which indicates a successful “reverse attack”. More details are provided in an article published in Nature by Chi Chen et al. [2] about the NASA press release. By analyzing the remote sensing data collected by NASA satellites from 2000 to 2017, researchers found an “unexpected joy”: The global green area “rose against the trend” by 5%, which is an equivalent area to that of the Amazon tropical rainforest. The move towards green development in China’s industry is becoming increasingly clear and relies on three steps: Step 1, emphasize the conservation of energy resources; Step 2, create a new path for the industrial coordination of the development of human resources, environmental protection and economic benefits; Step 3: create an ecological civilization focusing on the concept of green development.

At present, there are different perspectives in the academic research on achieving an ecological civilization in China. First, from a governance perspective, implementing rules and regulations and linking ecological evaluation with performance can play a positive role in the creation of an ecological civilization [3]. Research has shown that the growth of the tourism economy and urban and rural development in Beijing, Tianjin and Jilin can be achieved by focusing on both the economic and ecological benefits. Second, from the perspective of ecological compensation, addressing the needs of government and business, following the appropriate management model will be beneficial [4]. Third, from the perspective of information dissemination and managing public opinion, we examine three essential factors for dealing with environmental problems that may influence public opinion related to the creation of an ecological civilization. First, regarding reaction time and taking the “Kunming PX incident” as an example, it has been claimed that the government should address such incidents in a timely manner [5]. Second, considering the factor of locality, by analyzing the actual events of a village, a consideration of specific local features may be useful to promote the generation of collective behavior, reduce the risk of “free riding” behavior, promote a collective identity and accelerate the dissemination of information [6]. In addition, we consider the features of information dissemination by the government when dealing with group movements in response to environmental problems. Establishing a “trusting” relationship with the public has a synergistic effect on the success of crisis handling. Government policies, communication and response in the age of the internet are very important for effective governance.

In addition to promoting legal systems and policies, the abovementioned research shows that cooperation among interested parties, including the government, businesses and the public, can play a key role in the creation of an ecological civilization; the introduction of a multiagent cooperative governance model is an important development. Therefore, to improve decision-making for issues related to ecological civilizations, we propose increasing the number and types of decision makers.

### 1.2. Public Participation Mechanism

The new public management theory proposes to transform western governments [7] and compares the public to customers and government departments to beneficiaries [8]. G. P. Whitator believes that compared with the government and other service organizations providing a finished product to the society, it is better to introduce public participation so that the government and other service organizations can create a finished product that better meets the needs of the public [9]. Bovaird clearly pointed out that the traditional concept of public service design and management, without considering the cooperative relationship between multiple stakeholders, is no longer suitable for current needs. He constructed a conceptual framework of cooperative production including users and service society and listed some cases of enhancing cooperation and improving local services [10]. In the process of studying the disposal of abandoned nuclear power plants, Alan Bond et al. proposed that the reasons affecting public participation include whether public attitudes are taken seriously and adopted, the extent of public understanding of project information, the extent of project-related information disclosure provided by the government and whether the government has achieved transparent power utilization, etc. [11]. T. Meinhard Doelle and A. Hn Sinclair pointed out that the public should not only participate in the whole process of environmental evaluation but also advance the time of public participation to the early stage of evaluation [12]. For example, Britain’s famous “one-arm away “management model began in the 1840s. The model was set up by the Queen and royal assent to the semi-official bodies that carry out the country’s cultural policies: the National Entertainment Services Federation and the Music and Arts Council of England. Later, in order to further strengthen the government’s support for cultural undertakings, the government established the Ministry of Arts and Libraries; they stipulated that this department was aimed at developing art in the whole country and could grant funds to domestic cultural institutions on behalf of the government. Under the “one-arm away” management mode, the government does not directly manage cultural service behaviors, which enables the public and social organizations to have more independent rights and interests. The research on public participation in environmental protection is more about NIMBY, which means Not In My Back Yard. The public is more involved in the decision-making process of the government from the perspective of their own survival and safety. In the past 40 years of reform and opening up, China’s rapid economic take-off had brought unprecedented opportunities for urban development. With the rapid advancement of urbanization, China’s cities are in a high-quality development stage. During this process, the contradiction between urban development and a desirable living environment is highlighted. One manifestation of this is that the construction of urban infrastructure, especially garbage treatment plants, sewage treatment plants, mortuary and other NIMBY facilities, makes the surrounding residents feel uncomfortable, resulting in NIMBY problems such as the Xiamen PX incident and the Guangzhou Panyu garbage incineration plant incident [13]. 

To expand the set of decision makers, this paper examines the inclusion of public organizations and citizen representatives in decisions related to cultural tourism projects. Because the public has a strong interest in the development of cultural and tourism projects, understanding the public’s attitudes and opinions greatly impacts the results of decision-making around these issues. The concept of a “stakeholder” first appeared in 1708. The Stanford University Research Group defined the term “stakeholder” as an indispensable external force in the process of organizational development. Stakeholder theory has been expanded and applied to other fields [14]. Cleland was the first to extend stakeholder theory to research dealing with issues related to public projects. It is of profound practical and theoretical significance to properly integrate stakeholders into the decision-making system for public issues [15,16]. In the current environment, the structure of decision-making is closed, and the public, although having a direct interest in the outcome of the decision-making process, is excluded from the process. In this model, the public’s limited understanding of risks may increase the probability of protests by environmental groups [17]. Because the implementation of cultural and tourism projects has both social and economic implications, public opinion should be considered. Since the Rio Summit in 1992, promoting public participation in environmental decisions has been a common initiative in many countries. International scholars have highlighted the importance of a multi-governance model that includes public participation in policy decisions and have recognized public participation as a key success factor for environmental impact assessment in China. The public should be included as a collaborator for environmental decision-making, and public understanding and support should be sought to ensure the effectiveness of public engagement.

Stakeholder theory provides the scientific rationale for including the public in the decision-making process of creating an ecological civilization. Due to the gradual increase in awareness of the importance of public participation and the recent implementation of laws and policies regarding public participation in China, the influence of public groups’ opinions on handling environmental problems has increased [18]. Public participation is a behavior that depends on an individual’s level of education and self-interest. Education level can influence the perceptions of and the value placed on the environment, and the level of participation by the public in various forms of environmental governance depends on their assessment of their potential individual benefits and risks [19]. Incorporating different points of view helps reach an understanding of the interests of different entities during the creation of an ecological civilization [20]. Public participation in the process of creating an ecological civilization can promote awareness and coordinate multiple interests to form a “Pareto optimal” state in the realm of social governance. It can reinforce a sense of identity, promote the sense of belonging and empower individuals to participate in the creation of an ecological civilization [12].

Examining the example of developed countries and their evolving trends in environmental policies, it is clear that public participation enriches the information available for decision-making and incorporates the concerns of various stakeholders, thereby providing a more comprehensive approach to problem solving. Therefore, public participation decisions about environmental protection have become essential to the constructive management of governments. However, the integration of comments from public hearings and the litigation of violations of environmental protection law by citizens is not enough to effectively address the need for public participation [21,22]. Going beyond a preliminary level of public participation has been a challenge in the field of environmental protection, and it should be the public’s right to participate in the creation of an ecological civilization. The implementation of public participation requires a system of specific and feasible regulations true to the concept of green development [13]. The current legal system is not specific enough to promote the public’s right to participation. It only specifies high-level requirements and restrictions, making it difficult for citizens to exercise their rights. Therefore, in the case analysis section, we introduce the public representative model by which the public can effectively express their ideas.

### 1.3. Research Status Analysis on Large-Scale Group Decision-Making Problems

#### 1.3.1. Expression Tool of Decision Maker’s Evaluation Information

Some things in life can be quantified, such as company profits or number of papers published. However, other things cannot be assigned exact numerical values, such as the survival prospect of a company, the merit of a scholar or the evaluation of the advantages and disadvantages of the tourism environment. Because people are bound by rational thought, this can cause hesitation when making decisions. The concept of fuzzy numbers first proposed by American cybernetics expert Zadeh in 1965 broke away from the tradition of binary logic [23]. Ten years later, Zadeh introduced the concept of interval fuzzy numbers and the assignment of membership degree to an interval, which can be applied to practical problems [24]. In some practical applications, there will always be a certain degree of hesitation when making a judgment. For example, in elections, there are three types of voters: those who vote in support, those who vote against and those who are undecided and choose to abstain. To address this situation, the concept of an intuitionistic fuzzy number is proposed. Atanassov [25] proposed a mathematical conceptual framework for considering these three types of voters at the same time. Intuitionistic fuzzy numbers include the degrees of satisfaction, dissatisfaction and hesitation of decision makers, which closely reflect the attitude of decision makers in dealing with ambivalence and uncertainty. Based on the existing fuzzy set theory, combined with the advantages of interval fuzzy sets and intuitionistic fuzzy sets, the interval valued intuitionistic fuzzy number (IVIFN) was proposed [26] as a framework more in line with people’s inner process for decision-making. This approach appropriately uses intervals to express people’s various attitudes when making a decision: the degree of satisfaction (membership), the degree of dissatisfaction (non-membership) and the degree of uncertainty (hesitation). This concept was received with great enthusiasm by scholars in a variety of fields [27,28,29,30]. Since then, Atanassov et al. have paid attention to the properties of different fuzzy numbers [31,32,33,34,35]. With the rapid development of the methodology, practical applications could apply the possibility methods about different fuzzy sets [36,37]. At present, there have been many studies on interval intuitionistic fuzzy numbers and intuitionistic fuzzy number operators [38,39,40]. However, most of the studies explored the integration of data based on an algebraic approach, and few scholars have examined the problem from a geometric perspective. Interpreting data from a geometric perspective can be more intuitive and lead to insights into the meaning of data. Wan and Dong [41] introduced a method for comparing IVIFNs based on probability, defining the possibility degree of comparison between two IVIFNs. This paper assesses the geometric significance of IVIFN. By representing IVIFN with a rectangular coordinate system, we are able to analyze the area and center of gravity of the graph, as well as the limitations of its range. This is achieved by obtaining the conversion operator in order to convert it into IFN and further integrate and process the information of the newly obtained IFN. This method effectively frames the process of decision makers in a more intuitive way. 

#### 1.3.2. Research on LSGDM Methods

Due to the increasing complexity of practical problems, traditional group decision-making methods have been limited in their usefulness to some applications. Li [42] determined that when the number of decision makers is at least 20, it can be considered a LSGDM problem. Subsequently, a series of related studies on the LSGDM method were published [43,44,45,46]. The key elements of this methodology are to apply a scientific clustering algorithm, reasonably decompose the decision-making subject into small groups, integrate the most information for each small group and then summarize the information. At present, there are two main perspectives for evaluating clustering algorithms. One approach is to analyze the social network relationship of decision makers [47,48], with methods such as C-means clustering [47] or K-means clustering [48]. Another approach is to analyze the consistency of the decision information [49,50,51,52,53], aiming at obtaining maximum consensus-increasing by minimum cost. The ultimate goal of clustering algorithms is to classify the members of large groups into different smaller groups. Previous studies have shown that only considering the consistency of the initial evaluation of information and ignoring the social network relationships between decision makers may have a negative impact on the success of the final decision-making results [54,55]. The benefits of social analysis have been confirmed by some studies [56,57,58,59,60]. The internet has become a simple and convenient forum for decision makers to communicate with each other. Therefore, the social network relationship between decision makers has an impact on the results of individual evaluations. In this paper, we consider the trust relationship between decision makers before their evaluation, and so the concept of a trust score is introduced. This paper summarizes the relevant information for each decision maker at the beginning of the decision-making process. The decision maker first assigns a trust value [61] and a distrust value to other decision makers. Then, the trust relationship network is constructed based on the trust values, and the clustering algorithm is applied. The results of the clustering are adjusted based on their appropriateness. In order to analyze the trust and distrust values, we represent them geometrically by placing them in the rectangular coordinate system. This reflects the evaluation information of decision makers more clearly and intuitively.

The remainder of this paper is organized as follows. Section 2 provides basic definitions of trust relationships and of intuitionistic fuzzy numbers, whose geometric meaning has also been studied. The GN algorithm is used to deal with the directed graph problem. In addition, a novel large-scale group decision-making method is proposed. An illustrative example is discussed in Section 3. Section 4 compares the proposed method with a different method. Conclusions and directions for further research are discussed in Section 5.

## 2. Materials and Methods

In this section, two parts of the basic knowledge system and related expansion research will be described in detail.

### 2.1. Trust–Distrust Value

**Definition 1 [61].**  *A trust–distrust score* (t,d) *belongs to the set* [0,1]×[0,1] *, where* t *stands for the trust value,* d *stands for the distrust value. The space* $BL{}^{\circ}=\left({\left[0,1\right]}^{2},\leq_{t},\leq_{d},\neg \right)$ *is composed of a trust–distrust score set, trust ranking* $\left(\leq_{t}\right)$ *, knowledge level ranking* $\left(\leq_{k}\right)$ *and negative operator* (¬) *. All trust scores* $\left(t_{1},d_{1}\right)$ *and* $\left(t_{2},d_{2}\right)$ *(where* $0\leq t_{1},d_{1},t_{2},d_{2}\leq 1$ *) satisfy the following:*

$$\begin{array}{c} \left(t_{1},d_{1}\right)\leq_{t}\left(t_{2},d_{2}\right)\;\text{iff}\;t_{1}\leq t_{2}\;\text{and}\;d_{1}\geq d_{2}\\ \left(t_{1},d_{1}\right)\leq_{k}\left(t_{2},d_{2}\right)\;\text{iff}\;t_{1}\leq t_{2}\;\text{and}\;d_{1}\leq d_{2}\\ \neg \left(t_{1},d_{1}\right)=\left(d_{1},t_{1}\right) \end{array}$$

**Definition 2 [24].**  *The trust value and knowledge deficit associated with trust–distrust score* (t,d) *are defined as follows:*

TS(t,d)=t−dKD(t,d)=|1−t−d|

**Definition 3 [24].**  *Let* $\alpha_{1}=\left(t_{1},d_{1}\right)$ *and* $\alpha_{2}=\left(t_{2},d_{2}\right)$ *be two trust–distrust scores, their trust values and knowledge deficits are:* $TS_{1}=t_{1}-d_{1}$ *,* $TS_{2}=t_{2}-d_{2}$ *;* $KD_{1}=\left|1-t_{1}-d_{1}\right|$ *,* $KD_{2}=\left|1-t_{2}-d_{2}\right|$ *. Then, we have the following:*

*If* $TS_{1}≺TS_{2}$ *,* $\left(t_{1},d_{1}\right)≺\left(t_{2},d_{2}\right)$ *;*

*If* $TS_{1}≻TS_{2}$ *,* $\left(t_{1},d_{1}\right)≻\left(t_{2},d_{2}\right)$ *;*

*If*$TS_{1}=TS_{2}$*, and if*$KD_{1}≺KD_{2}$*,*$\left(t_{1},d_{1}\right)≻\left(t_{2},d_{2}\right)$*; if*$KD_{1}≻KD_{2}$*,*$\left(t_{1},d_{1}\right)≺\left(t_{2},d_{2}\right)$*; and if*$KD_{1}=KD_{2}$*,*$\left(t_{1},d_{1}\right)=\left(t_{2},d_{2}\right)$.

*We define “* 1−KD *” as the knowledge. Without loss of generality, the smaller the knowledge deficit, the greater the degree of knowledge. When the knowledge deficit is the same for two trust–distrust scores, the greater the trust score, and vice versa.*

**Example** **1.** *The properties of Definition 3 will fail when comparing some trust–distrust scores. That is, any given two different trust–distrust scores cannot be distinguished based on trust score and knowledge deficit. For example, for trust–distrust scores*$\alpha_{1}=\left(0.55,0.35\right)$*and*$\alpha_{2}=\left(0.65,0.45\right)$, $TS_{1}=TS_{2}=0.2$, $KD_{1}=KD_{2}=0.1$*. However, according to Definition 1,*$\alpha_{1}{<}_{k}\alpha_{2}$*. Thus, there is no single operator to integrate trust value and distrust value. They can be transformed into a value that can be whose comparison is more intuitive. Next, we will explain the broader reasons for this phenomenon.*

**Lemma** **1.** *For trust–distrust score* $\alpha_{i}=\left(t_{i},d_{i}\right)$ *, where* i=1,2 *,* $TS_{1}=TS_{2}$ *and* $KD_{1}=KD_{2}$ *when* $t_{1}+d_{2}=1$ *,* $t_{2}+d_{1}=1$ *. In this situation, the trust–distrust scores cannot be distinguished.*

**Proof.**  If $TS_{1}=TS_{2}$, $t_{1}-d_{1}=t_{2}-d_{2}=x$, $t_{1}=x+d_{1}$, $t_{2}=x+d_{2}$, $KD_{1}-KD_{2}=\left|1-t_{1}-d_{1}\right|-\left|1-t_{2}-d_{2}\right|$

To compare $KD_{1}$ and $KD_{2}$, there are two cases:

Case 1:

$$\begin{array}{ll} KD_{1}-KD_{2} & =\left(1-t_{1}-d_{1}\right)-\left(1-t_{2}-d_{2}\right)\\ & =1-t_{1}-d_{1}-1+t_{2}+d_{2}\\ & =t_{2}-t_{1}+d_{2}-d_{1}\\ & =\left(x+d_{2}\right)-\left(x+d_{1}\right)+d_{2}-d_{1}\\ & =2\left(d_{2}-d_{1}\right) \end{array}$$

Let $KD_{1}-KD_{2}=0$, obtaining $2\left(d_{2}-d_{1}\right)=0$, $d_{1}=d_{2}$.

Because $t_{1}=x+d_{1}$, $t_{2}=x+d_{2}$, obtaining $t_{1}=t_{2}$.

Case 2:

$$\begin{array}{ll} KD_{1}-KD_{2} & =\left(1-t_{1}-d_{1}\right)-\left(-\left(1-t_{2}-d_{2}\right)\right)\\ & =1-t_{1}-d_{1}+1-t_{2}-d_{2}\\ & =2-\left(t_{1}+t_{2}+d_{1}+d_{2}\right)\\ & =2-\left(x+d_{1}+x+d_{2}+d_{1}+d_{2}\right)\\ & =2-2\left(x+d_{1}+d_{2}\right) \end{array}$$

**Figure 1:**
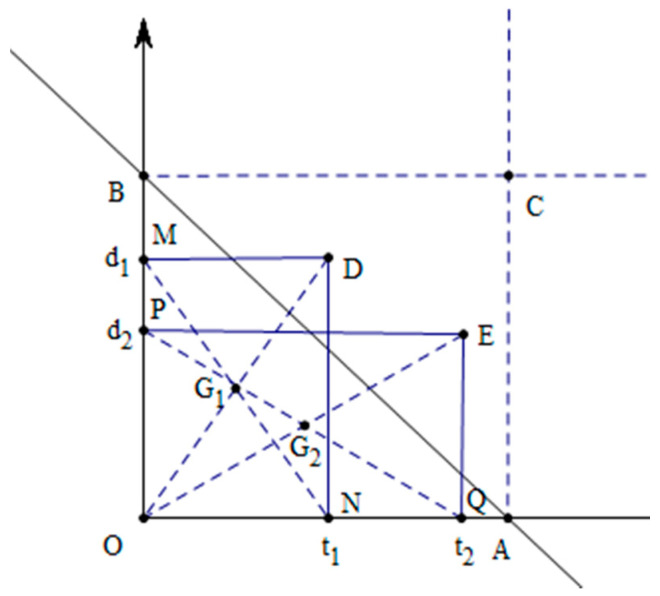
Geometric meaning of the trust–distrust score.

Let $KD_{1}-KD_{2}=0$, giving $2-2\left(x+d_{1}+d_{2}\right)=0$, $x+d_{1}+d_{2}=1$,$d_{1}+d_{2}=1-x\in \left[0,1\right]$, then $t_{1}-d_{1}+d_{1}+d_{2}=1$, i.e., $t_{1}+d_{2}=1$. Similarly, $t_{2}+d_{1}=1$ can be obtained. That is, the trust–distrust values satisfying such characteristics cannot be identified by functions KD and TS. Taking the two trust–distrust scores in Example 1 as an example, for $\alpha_{1}$ and $1-t_{1}-d_{1}=0.1$, there is an uncertainty of 0.1 units; for $\alpha_{2}$ and $1-t_{1}-d_{1}=-0.1$, there is a knowledge overflow of 0.1 units. We can clarify this through an analysis of extreme cases. From Figure 1, the decision maker assigns the trust–distrust values, and trust–distrust set can be represented as a point on line AB, when $1-t_{1}-d_{1}=0$; this point is the origin meaning that the net opinion of the decision maker is neither trust nor distrust. When $1-t_{1}-d_{1}=1$, $t_{1}=0$, $d_{1}=0$, this point C and indicates a high degree of trust, and, similarly, when $1-t_{1}-d_{1}=-1$, $t_{1}=1$ and $d_{1}=1$, this indicates a high degree of distrust. It can be seen that the smaller the value of ${\left|1-t_{1}-d\right|}_{1}$ is, the clearer the decision result, and the larger the value of $\left|1-t_{1}-d_{1}\right|$, the fuzzier the decision result. □

Suppose $\alpha_{1}=\left(t_{1},d_{1}\right)$ and $\alpha_{2}=\left(t_{2},d_{2}\right)$ are two trust scores, shown in Figure 1 by points D and E. The coordinates of A, B and C are A=(1,0), B=(0,1) and C=(1,1). The points $G_{1}$ and $G_{2}$ are the gravity geometric centers of rectangles ▭ONDM and ▭OQEP.

**Definition** **4.** *The decision information area integration operator of*(t,d,1−t,1−d)*is*s=td.

Property of area integration operator s=td:

Case 1: $1-t_{1}-d_{1}=0$. The trust boundary of the decision results is clear, so the decision information area is calculated as s=t(1−t), where the larger the value of t, the larger the value of s, when $t\in \left[0,\frac{1}{2}\right]$; and the larger the value of t, the smaller the value of s, when $t\in \left[\frac{1}{2},1\right]$. In both cases, the larger values of s represent higher accuracy.

Case 2: $1-t_{1}-d_{1}\neq 0$. Smaller values indicate higher accuracy, that is, the decision result represented by area ΔOAB is better than that of area ΔCAB.

**Theorem** **1.** *For any two trust–distrust scores*$\alpha_{i}=\left(t_{i},d_{i}\right)$*, where*i=1,2*,*$\alpha_{1}=\alpha_{2}$*, when*$TS_{1}=TS_{2}$*,*$KD_{1}=KD_{2}$*and*$s_{1}=s_{2}$*, where*$s_{i}=t_{i}d_{i}$*,*i=1,2.

**Proof.**  Because $\alpha_{1}=\left(t_{1},d_{1}\right)$, $\alpha_{2}=\left(t_{2},d_{2}\right)=\left(1-d_{1},1-t_{1}\right)$, $s_{1}=t_{1}d_{1}$, $s_{2}=\left(1-t_{1}\right)\left(1-d_{1}\right)=1-t_{1}-d_{1}+t_{1}d_{1}$. When $s_{1}=s_{2}$, i.e., $1-t_{1}-d_{1}+t_{1}d_{1}=t_{1}d_{1}$, $1-t_{1}-d_{1}=0$, $t_{1}+d_{1}=1$, $t_{1}=1-d_{1}=t_{2}$. Similarly, $d_{1}=d_{2}$. □

Based on the information area integration operator defined in Theorem 1, Theorem 2 is presented.

**Theorem** **2.** *Let* $\alpha_{1}=\left(t_{1},d_{1}\right)$ *and* $\alpha_{2}=\left(t_{2},d_{2}\right)$ *be two trust–distrust scores; then their trust values:* $TS_{1}=t_{1}-d_{1}$ *,* $TS_{2}=t_{2}-d_{2}$ *, their knowledge deficits:* $KD_{1}=\left|1-t_{1}-d_{1}\right|$ *,* $KD_{2}=\left|1-t_{2}-d_{2}\right|$ *, the following conclusions are obtained:*

*If* $TS_{1}<TS_{2}$ *,* $\left(t_{1},d_{1}\right)≺\left(t_{2},d_{2}\right)$ *;*

*If* $TS_{1}>TS_{2}$ *,* $\left(t_{1},d_{1}\right)≻\left(t_{2},d_{2}\right)$ *;*

*If*$TS_{1}=TS_{2}$*, and if*$KD_{1}<KD_{2}$*, then*$\left(t_{1},d_{1}\right)≻\left(t_{2},d_{2}\right)$*; and if*$KD_{1}>1-KD_{2}$*, then*$\left(t_{1},d_{1}\right)≺\left(t_{2},d_{2}\right)$*; and if*$KD_{1}=KD_{2}$*,*$\left(t_{1},d_{1}\right)>\left(t_{2},d_{2}\right)$*when*$s_{1}<s_{2}$*;*$\left(t_{1},d_{1}\right)<\left(t_{2},d_{2}\right)$*when*$s_{1}>s_{2}$*;*$\left(t_{1},d_{1}\right)=\left(t_{2},d_{2}\right)$*when*$s_{1}=s_{2}$.

Definition 1 only defines a comparative relationship for sorting, without providing a specific numerical operator to apply. Therefore, this paper proposes the following trust–distrust score decision information aggregation (IA) operator:

(1) $$IA={\left({\left(t-d\right)}^{2}+{\left(t+d\right)}^{2}+\left(1-td\right)\right)}^{\frac{1}{2}}$$

by analyzing its geometric properties, where larger values of IA represent higher trust in response.

### 2.2. Social Network Analysis Method Based on Decision Information

Social network analysis examines the relationship between decision makers. Each point represents a decision maker, and the line between any two decision makers represents their social network relationship. In this paper, the degree of trust between decision makers is expressed by their network nodes and their relationship, and the definition of expression of trust is given below.

A weighted network is introduced, and then cluster analysis is performed based on the trust between decision makers in the network. First, the trust between decision makers is analyzed and clustered. The expression of trust is shown in Table 1 below. The detailed evaluation results are shown in Appendix A. The decision makers are randomly assigned a number, and these numbers are summarized. Decision makers assign a trust value to other decision makers in the network if there is a social network relationship between them; otherwise they do not assign a trust value. There are three possible scenarios: both decision makers assign each other a trust value; only one decision maker believes that there is a social network relationship and so only one trust value is assigned; there is no network relationship between them and neither decision maker assigns a trust value to the other. Trust relationships missing at least one trust value are denoted by (0,0). An example is given in the following table: the first and second columns represent the decision makers in each relationship pair, the third column represents the trust value assigned by the decision maker in the first column to the decision maker in the second column, the fourth column represents the trust value assigned by the decision maker in the second column to the decision maker in the first column, the fifth column is the average trust value, and the sixth column is the average accuracy. It is calculated by operators (2) and (3), respectively.

(2) $$O_{1}=\left(\left(t_{12}+t_{21}\right)-\left(d_{12}+d_{21}\right)\right)/2$$

(3) $$O_{2}=\left(\left(t_{12}+t_{21}\right)+\left(d_{12}+d_{21}\right)\right)/2$$

**Figure 2:**
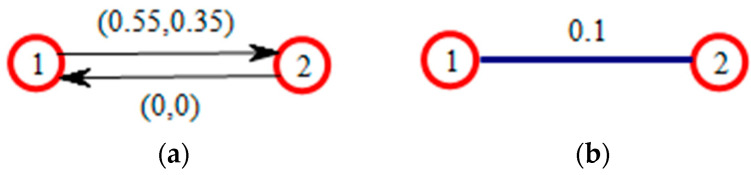
This is a figure about the trust relationship between two decision makers. (**a**) shows that the trust–distrust degree from decision maker 1 to decision maker 2 is (0.55, 0.35), and the trust–distrust degree from decision maker 2 to decision maker 1 is (0, 0). (**b**) shows that the average trust degree between decision maker 1 and decision maker 2 is 0.1 without direction.

The evaluation results are represented by the social network diagram, which is shown in Figure 2.

**Definition 5 [62].**  Edge betweenness is defined as the proportion of the total number of shortest paths in the network passing through the edge.

A descriptive function called modularity [63] is used to measure the aggregating result, defined by Equation (4), where $s_{i,in}$ is the edge numbers of node i and the other nodes in community C. The higher the modularity is, the better the aggregating results are.

(4) $$\Delta Q=\left[\frac{W_{C}+s_{i,in}}{2W}-{\left(\frac{S_{C}+s_{i}}{2W}\right)}^{2}\right]-\left[\frac{W_{C}}{2W}-{\left(\frac{S_{C}}{2W}\right)}^{2}-{\left(\frac{S_{i}}{2W}\right)}^{2}\right]$$

Under the Gewen-Newman (GN) algorithm [64], according to the edge betweenness centrality (EBC) value of the graph, the community in the graph is found by iteratively removing the edges of the graph. The edge with the greatest EBC is removed first. In this paper, the GN algorithm is applied to a weighted network, and its steps are as follows: 

Procedure 1

**Step 1.**  Calculate the number of edge intermediaries of all connected edges in the network relative to the source node;
**Step 2.**  Divide the edge intermediary number of each edge by its weight value to obtain the edge weight ratio of each edge;
**Step 3.**  Delete the edge with the highest edge weight ratio;
**Step 4.**  Repeat steps 1, 2 and 3;
**Step 5.**  Once no more edges exist in the network, the last generated split tree is taken as the divided community.

### 2.3. IVIFN and an Extension Distance Function

**Definition 6 [25].** 
$U=\left\{\left(x,\mu_{U}^{x},\nu_{U}^{x}\right)|\mu_{U}^{x},\nu_{U}^{x}\in R^{+}\right\}$ *is an intuitionistic fuzzy set defined in* $R^{+}$ *, satisfied* $\mu_{U}^{x}\in \left[0,1\right]$ *,* $\nu_{U}^{x}\in \left[0,1\right]$ *,* $\mu_{U}^{x}+\nu_{U}^{x}\in \left[0,1\right]$ *, where* $\mu_{U}^{x}$ *and* $\nu_{U}^{x}$ *represent the membership degree and non-membership degree, respectively.* $\pi_{U}^{x}=1-\left(\mu_{U}^{x}+\nu_{U}^{x}\right)$ *is called the hesitant degree.*

Intuitionistic fuzzy number contains three aspects of information: the degree of satisfaction (membership), the degree of dissatisfaction (non-membership) and the degree of uncertainty (hesitant degree). These numbers are best suited for dealing with problems with strong fuzziness and uncertainty. IVIFN is introduced when dealing with more complicated problems.

**Definition 7 [26].** 
$A=\left\{\langle x,u_{A}\left(x\right),v_{A}\left(x\right)\rangle |x\in X\right\}$ *is an interval valued intuitionistic fuzzy set defined in X,* $u_{A}^{x}=\left[a,b\right]:\;X\to L\left[0,1\right],\;\forall x\in X$ *,* ∀x∈X *;* $v_{A}^{x}=\left[c,d\right]:\;X\to L\left[0,1\right],\;\forall x\in X$ *,* b+d≤1 *,* ∀x∈X *; where* $u_{A}^{x}$ *and* $v_{A}^{x}$ *represent the membership degree and non-membership degree, respectively.* $\pi_{A}^{x}=\left[1-b-d,1-a-c\right]$ *is called the hesitant degree.*

**Figure 3:**
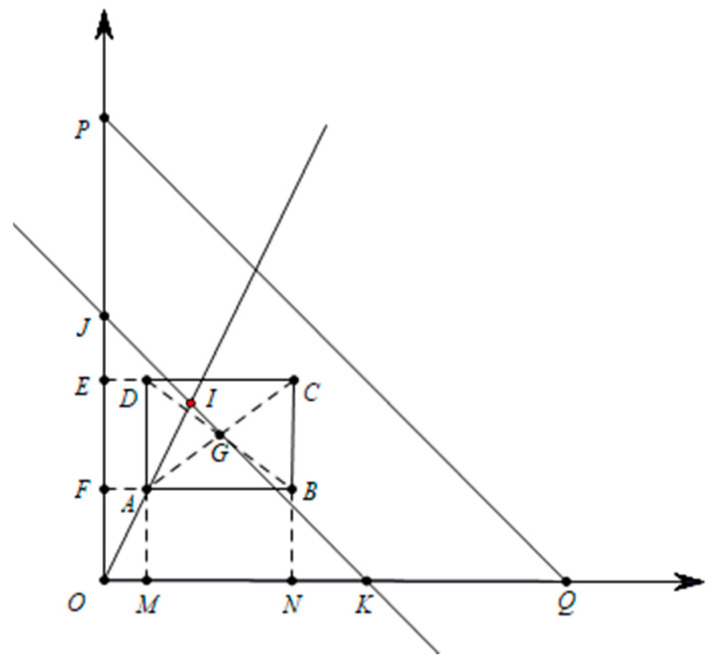
Geometric meaning of interval intuitionistic fuzzy numbers.

The IVIFN is written by α=〈[a,b],[c,d]〉 for simplicity. As shown in Figure 3, α is shown in the rectangular coordinate system. The coordinate values of each point are: A:(a,c), B:(b,c), C:(b,c), D:(a,d), P:(0,1), Q:(1,0). M, N, E and F are the projection points of points A, B, C and D on the coordinate axis. Point G is the center of gravity of rectangle ABCD. 

The straight line $L_{JK}$ is parallel to the straight line $L_{PQ}$ and passes through the center of gravity of the rectangle ABCD. The intersection of line $L_{OA}$ and line $L_{JK}$ is recorded as I, which is the optimal point representing interval intuitionistic fuzzy numbers. Therefore, the slope of $L_{JK}$ is −1. The coordinates of point *G* are $\left(a+\frac{b-a}{2},c+\frac{d-c}{2}\right)$. The straight line can be represented as follows:

$$L_{JK}:\;y=-x+\frac{a+b+c+d}{2}$$

The line $L_{OA}$ through points O and A, and its slope is $\frac{c}{a}$. Then the line $L_{OA}$ can be expressed as follows:

$$L_{OA}:\;y=\frac{c}{a}x$$

I is the intersection of $L_{OA}$ and $L_{JK}$, which represents the most likely point for IVIFN 〈[a,b],[c,d]〉. The coordinates could be calculated, I: $\left(\frac{\left(a+b+c+d\right)a}{2\left(a+c\right)},\frac{\left(a+b+c+d\right)c}{2\left(a+c\right)}\right)$, where a+c≠0. It is reasonable to state that the larger the area of the rectangle □ABCD to the triangle △OPQ is, the more uncertain it is. The uncertainty ratio R

$$R=\frac{\left(b-a\right)\times \left(d-c\right)}{1/2}=2\left(b-a\right)\left(d-c\right)$$

**Definition** **8.** *An INIFN* α=〈[a,b],[c,d]〉 *can be transformed to an IFN-form* β=(μ,ν) *based on the following equation:*

(5) $$\beta =\left(\mu ,\nu \right)=\left(\frac{a\left(a+b+c+d\right)(1-R)}{2\left(a+c\right)},\frac{c\left(a+b+c+d\right)(1-R)}{2\left(a+c\right)}\right)$$

*where* R *is the uncertainty ratio.*

The function between the new form two intuitionistic fuzzy numbers $\beta_{1}=\left(\mu_{1},\nu_{1}\right)$ and $\beta_{2}=\left(\mu_{2},\nu_{2}\right)$ is given as follows:

(6) $$d\left(\beta_{1},\beta_{2}\right)={\left[\frac{1}{5}\left({\left|\mu_{1}-\mu_{2}\right|}^{2}+{\left|\nu_{1}-\nu_{2}\right|}^{2}+{\left|H_{1}-H_{2}\right|}^{2}+{\left|C_{1}-C_{2}\right|}^{2}+{\left|S_{1}-S_{2}\right|}^{2}\right)\right]}^{\frac{1}{2}}$$

where $H_{i}=1-\mu_{i}-\nu_{i}$, i=1,2 stands for the hesitant degree of the IFN; $C_{i}=\mu_{i}+\nu_{i}$, i=1,2 stands for the certainty degree of the IFN; and $S_{i}=\mu_{i}\nu_{i}$ stands for the uncertainty area determined by these four points: $\left(\mu_{i},\nu_{i}\right)$,$\left(0,\nu_{i}\right)$,$\left(\mu_{i},0\right)$,(0,0).

**Theorem** **3.** *Let* $\alpha_{i}=\langle \left[a_{i},b_{i}\right],\left[c_{i},d_{i}\right]\rangle$ *be an IVIFN,* i=1,2 *. The number* $\beta_{i}=\left(\mu_{i},\nu_{i}\right)$ *obtained by Equation (5) is an IFN. The distance measure* $d\left(\beta_{1},\beta_{2}\right)$ *satisfies the following properties.*

*(1)*$0\leq d\left(\beta_{1},\beta_{2}\right)\leq 1$*(2)*$d\left(\beta_{1},\beta_{2}\right)=1$ iff $\beta_{1}=\beta_{2}$ *(3)* $d\left(\beta_{1},\beta_{2}\right)=d\left(\beta_{2},\beta_{1}\right)$

**Proof.**  First, as $a_{i},b_{i},c_{i},d_{i},R\in \left[0,1\right]$, $a_{i}+b_{i}+c_{i}+d_{i}\in \left[0,2\right]$,$\mu_{i}=\frac{a_{i}\left(a_{i}+b_{i}+c_{i}+d_{i}\right)(1-R)}{2\left(a_{i}+c_{i}\right)}\in \left[0,1\right]$, $\nu_{i}=\frac{c_{i}\left(a_{i}+b_{i}+c_{i}+d_{i}\right)(1-R)}{2\left(a_{i}+c_{i}\right)}\in \left[0,1\right]$ $\mu_{i}+\nu_{i}=\frac{a\left(a+b+c+d\right)(1-R)}{2\left(a+c\right)}+\frac{c\left(a+b+c+d\right)(1-R)}{2\left(a+c\right)}=\frac{\left(a+b+c+d\right)(1-R)}{2}\in \left[0,1\right]$. Therefore, the number $\beta_{i}=\left(\mu_{i},\nu_{i}\right)$ obtained by Equation (5) is an IFN.

Second, we prove the three properties.

(1) Based on the definition of $\mu_{i},\nu_{i},\mu_{i}+\nu_{i}\in \left[0,1\right]$, we can determine that ${\left|\mu_{1}-\mu_{2}\right|}^{2}\in \left[0,1\right]$, ${\left|\nu_{1}-\nu_{2}\right|}^{2}\in \left[0,1\right]$, ${\left|H_{1}-H_{2}\right|}^{2}\in \left[0,1\right]$, ${\left|C_{1}-C_{2}\right|}^{2}\in \left[0,1\right]$, ${\left|S_{1}-S_{2}\right|}^{2}\in \left[0,1\right]$, then $d\left(\beta_{1},\beta_{2}\right)\in \left[0,1\right]$.
(2) If $d\left(\beta_{1},\beta_{2}\right)=0$, then consider the definition of $d\left(\beta_{1},\beta_{2}\right)$ and
  $$\left\{ \begin{array}{l} {\left|\mu_{1}-\mu_{2}\right|}^{2}\geq 0\\ {\left|\nu_{1}-\nu_{2}\right|}^{2}\geq 0\\ {\left|H_{1}-H_{2}\right|}^{2}\geq 0\\ {\left|C_{1}-C_{2}\right|}^{2}\geq 0\\ {\left|S_{1}-S_{2}\right|}^{2}\geq 0 \end{array}\right.$$
  and obtain
  $$\left\{ \begin{array}{l} {\left|\mu_{1}-\mu_{2}\right|}^{2}=0\\ {\left|\nu_{1}-\nu_{2}\right|}^{2}=0\\ {\left|H_{1}-H_{2}\right|}^{2}=0\\ {\left|C_{1}-C_{2}\right|}^{2}=0\\ {\left|S_{1}-S_{2}\right|}^{2}=0 \end{array}\right.$$
  The above equations mean $\mu_{1}=\mu_{2}$,$\nu_{1}=\nu_{2}$, i.e., $\beta_{1}=\beta_{2}$.
(3) It is obvious that $d\left(\beta_{1},\beta_{2}\right)=d\left(\beta_{2},\beta_{1}\right)$. □

**Example** **2.** *There are two kinds of patterns*$P_{1}$*and*$P_{2}$*, which are represented by IFNs*$\tilde{P_{1}}=\left\{\langle x_{1},0.6,0.25\rangle ,\langle x_{2},0,0.25\rangle ,\langle x_{1},0.3,0.25\rangle \right\}$*,*$\tilde{P_{2}}=\left\{\langle x_{1},0.1,0.75\rangle ,\langle x_{2},0.15,0.1\rangle ,\langle x_{1},0.2,0.35\rangle \right\}$*in the universe of*$X=\left\{x_{1},x_{2},x_{3}\right\}$*. A different pattern is represented by*$\tilde{Q}=\left\{\langle x_{1},0,0.15\rangle ,\langle x_{2},0.325,0.425\rangle ,\langle x_{1},0.2,0.25\rangle \right\}$*. The next step is to determine which of the two patterns is closest to*$\tilde{Q}$*. Different authors have proposed different methods for accomplishing this. The results of classification based on various methods are shown in*Table 2*. From*Table 2*, the distance measure of Reference* [27] *could not be determined; the other 3 methods agree that pattern Q is closest to the P_2_ but differ in the specific distances between the patterns. Comparing the different values of D_1_(P_1_, Q) and D_2_(P_1_, Q), we find that the results are the same.*

### 2.4. A Weighting Algorithm Based on the Novel Distance Measure

Suppose there are m alternatives $A_{i}$, i=1,2,…,m. In this study, IVIFNs are used to describe the evaluation results given by decision experts $DM_{d}$, d=1,2,…,D, according to the *n* criteria $C_{j}$, j=1,2,…,n. The evaluating result is $\alpha_{ij}^{d}=\langle \left[a_{ij}^{d},b_{ij}^{d}\right],\left[c_{ij}^{d},d_{ij}^{d}\right]\rangle$. Among the alternatives, if the difference between the result of evaluation assigned by a decision maker based on a certain criterion is small, it indicates that the evaluation standard has a weak ability to distinguish among alternatives, and the evaluation standard should be assigned a lower weight. If there is a large difference between results of evaluation based on a given criterion, it indicates that the evaluation standard has a strong ability to distinguish among alternatives and should be assigned a higher weight. Therefore, based on the comprehensive distance formula $d(\beta_{ij}^{d},\beta_{kj}^{d})$ for calculating the difference degree between interval intuitionistic fuzzy numbers given in Formula (4), the following model is constructed from the perspective of criteria:

(7) $$w\left(C_{j}^{d}\right)=\frac{\sum_{i=1}^{m}\sum_{k=1}^{m}\sum_{1\leq i<k\leq m}d(\beta_{ij},\beta_{kj})}{\sum_{j=1}^{n}\sum_{i=1}^{m}\sum_{k=1}^{m}\sum_{1\leq i<k\leq m}d(\beta_{ij},\beta_{kj})}$$

The decision maker’s weight is determined by his or her difference degree for different alternatives, which is calculated by Equation (8):

(8) $$w(DM_{j}^{d})=\frac{\sum_{j=1}^{n}\sum_{i=1}^{m}\sum_{k=1}^{m}\sum_{1\leq i<k\leq m}d(\beta_{ij},\beta_{kj})}{\sum_{d=1}^{D}\sum_{j=1}^{n}\sum_{i=1}^{m}\sum_{k=1}^{m}\sum_{1\leq i<k\leq m}d(\beta_{ij},\beta_{kj})}$$

### 2.5. Construction of the LSGDM System Considering Decision Makers Trust–Distrust Relations

**Figure 4:**
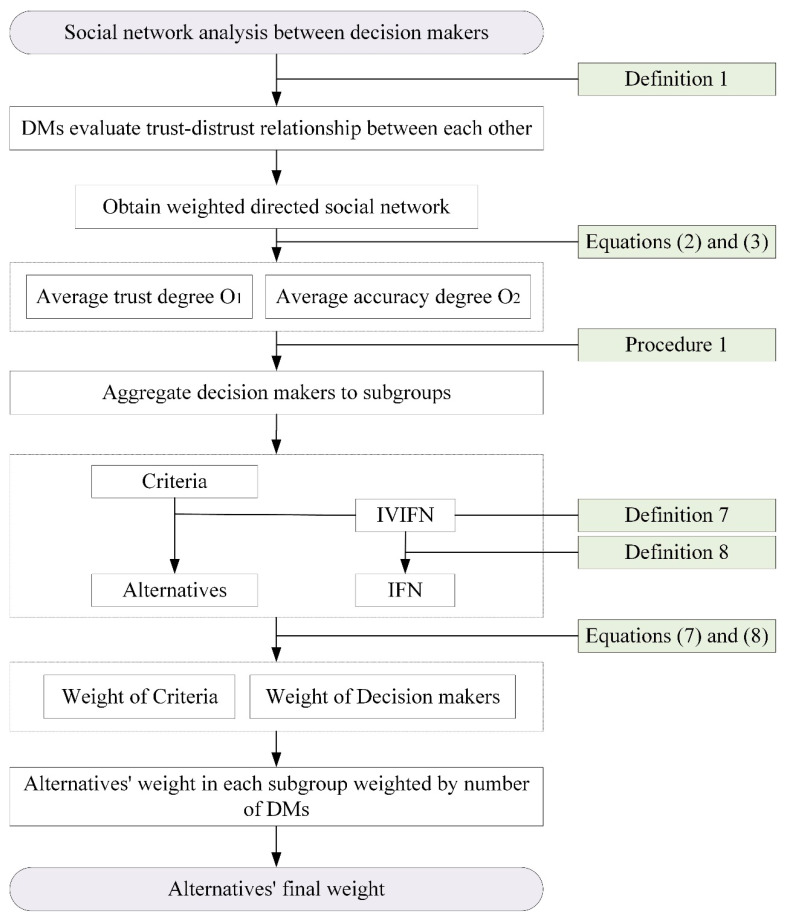
Process of the proposed method.

Based on the analysis of Section 2.1, Section 2.2 and Section 2.3, the procedure of LSGDM system with DM’s trust–distrust relations, are shown in Figure 4, details stated in Procedure 2.

Procedure 2

Step 1. Obtain the trust–distrust scores between decision makers based on Definition 1. Use Equations (2) and (3) to calculate the weight between decision makers from the trust–distrust network. 

Step 2. Apply Procedure 1 to aggregate all decision makers S to different subgroups $S_{p}$, where $S=\left\{S_{1},S_{2},\cdots ,S_{P}\right\}$.

Step 3. Determine alternatives set $A=\left\{A_{1},A_{2},\cdots ,A_{N}\right\}$ and criteria set $C=\left\{C_{1},C_{2},\cdots ,C_{M}\right\}$. 

Step 4. Original evaluations are described by IVIFNs by Definition 7. Use Equation (5) from Definition 8 to change the IVIFN to IFN.

Step 5. Apply Equation (7) to calculate the criteria’s weights $w\left(C_{j}^{d}\right)$ and Equation (8) to calculate the decision makers’ weights $w(DM_{j}^{d})$. 

Step 6. Calculate each alternative’s weight in each subgroup:$w(A_{i}^{Q_{p}})=\sum_{j=1}^{m}\sum_{d=1}^{Q_{p}}w(DM_{j}^{d})w\left(C_{j}^{d}\right)$, where $Q_{p}=\#\left\{S_{p}\right\}$.

Step 7. Each subgroup’s weight is determined by the number of decision makers. Then, the alternative final rankings are obtained by $w(A_{i})=\sum_{Q_{p}=1}^{P}w(A_{i}^{Q_{p}})w\left(Q_{p}\right)$, where $w\left(Q_{p}\right)=\frac{\#\left\{S_{p}\right\}}{\sum_{p=1}^{P}\#\left\{S_{p}\right\}}$. 

## 3. Case Analysis

### 3.1. Construction of Evaluation Mechanism of Cultural Tourism

In order to develop the economic level of a village located in Hebei Province, China, it is decided to build an evaluation index system from the following four perspectives based on the historical development and the actual situation of the region, combined with the alternatives given in Appendix B. We can get the following conclusions: First, we determined the trust relationship among the public groups participating in the survey. In our model, each individual is represented by a point. Trust values are assigned, and the group-directed weighted network structure is obtained. Then, the analysis dimension of the evaluation index system is determined. Based on the analysis of questionnaire responses, the evaluation is carried out based on the following four dimensions:

$C_{1}$: Natural environment dimension, including trees, vegetation, mountains and rivers;
$C_{2}$: Social environment dimension, including public security, culture, morality and history;
$C_{3}$: Economic environment dimension, including regional GDP index, infrastructure improvement level, etc.;
$C_{4}$: Stakeholder dimension, including the folk customs of residents in the region.

Based on the recommendation of experts, four cultural tourism projects have been proposed:

$A_{1}$: Restore a culturally important historical town;
$A_{2}$: Develop eco-tourism focusing on farm specialties;
$A_{3}$: Promote leisure tourism based on the weather;
$A_{4}$: Build a new fashion tourism village.

### 3.2. Decision Process Depending on the Proposed Method

**Figure 5:**
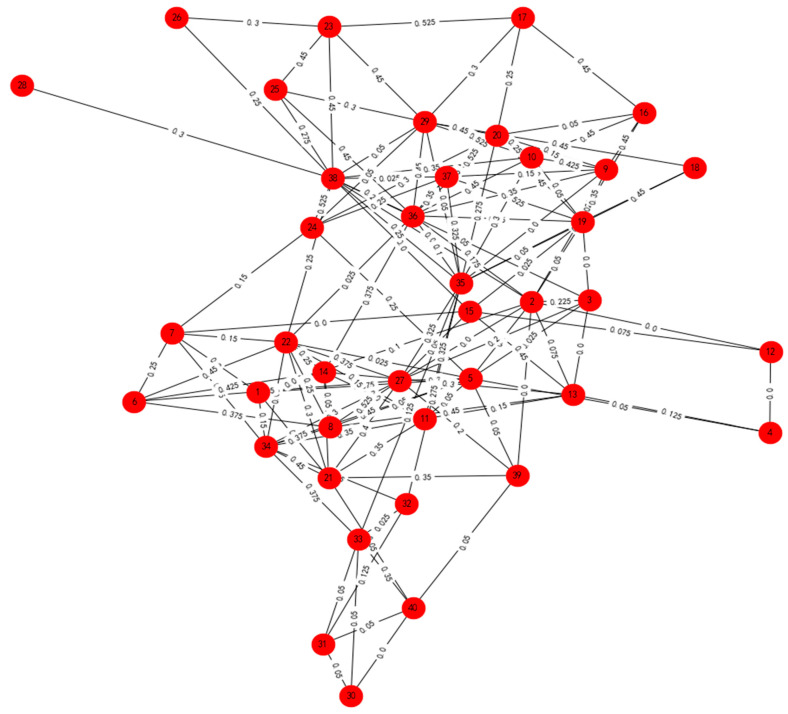
The original trust degree between decision makers.

**Figure 6:**
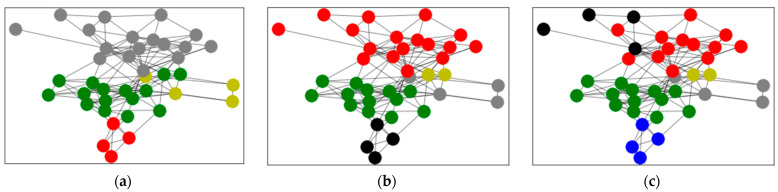
The aggregation result based on average trust degree. (**a**) shows the aggregation result of 4 subgroups; (**b**) shows the aggregation result of 5 subgroups; (**c**) shows the aggregation result of 6 subgroups.

**Figure 7:**
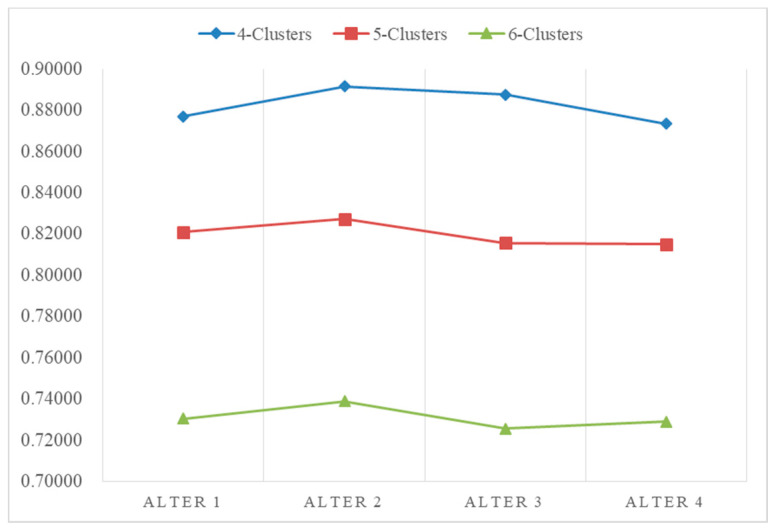
Ranking results of different numbers of clusters.

Forty decision makers were invited to evaluate the project proposals. Based on the literature review and empirical research, the decision makers included 10 people from relevant government departments, 10 expert scholars (the research of these experts comes from the field of economic management. Their research interests include regional economic analysis, marketing analysis, human resource management, cultural tourism management and supply chain management), 10 representatives of the service industry and representatives of public groups (including 5 representative local citizens and 5 individuals from nearby cities). These 40 decision makers were assigned numbers. The decision results are given in Table A1 from Appendix A. Then, the 40 decision makers provided their evaluations of the four alternatives by IVIFNs, which are shown in Appendix C. Applying Procedure 2, the aggregated and ranked results are shown in Table 3 and Table 4 and Figure 5, Figure 6 and Figure 7.

Based on Equation (3), clustering was applied based on the average trust degree. After calculating the average trust degree of all network relationships, the weighted network diagram was obtained as shown in Figure 5. Applying Procedure 1, the weighted network graph was clustered and analyzed to obtain the clustering results shown in Figure 6. Figure 6a shows the aggregation result of four subgroups, where the grey points stand for Group A, the green points stand for Group B, the yellow points stand for Group C and the red points stand for Group D. Figure 6b shows the aggregation result of five subgroups, where the red points stand for Group A, the green points stand for Group B, the grey points stand for Group C, the black points stand for Group D and the yellow points stand for Group E. Figure 6c shows the aggregation result of six subgroups, where the green points stand for Group A, the red points stand for Group B, the grey points stand for Group C, the black points stand for Group D, the bule points stand for Group E and the yellow points stand for Group F. When the classification results are poor, the evaluation accuracy can be considered. The aggregated results for the 40 decision makers can be found in Table 3, where the numbers indicate the order of the decision makers. From Table 3, we find that the subgroups were different. With an increase in the number of subgroups, the clustering results became less clear.

From the definition of modularity, it can be seen that the modularity value mainly depends on the community allocation C of nodes in the network, that is, the community division of the network. It can be used to quantitatively measure the quality of network community division. The closer the modularity value is to 1, the stronger the community structure divided by the network, that is, the better the division quality. Therefore, the optimal network community division can be obtained by maximizing the modularity Q. Ranking results of different numbers of clusters are shown in the following Table 4. Depending on the definition of modularity, aggregating the decision makers into five clusters yielded the highest modularity, indicating the greatest aggregated result. Figure 6 shows the results of the ranking alternatives. It can be seen from these results that the ranked order of alternatives obtained by different cluster methods were not the same. However, it was determined that the optimal option is Alternative $A_{2}$. Generally, we focus on the clustering results that have the highest modularity. Therefore, based on the tradition of fruit farming in the town, the project of developing farming specialty cultural tourism was selected. Figure 7 shows the ranking of the alternatives, and the overall ranking results were the same. The best choice was indicated to be Alternative $A_{2}$.

## 4. Comparisons between Different Methods

**Figure 8:**
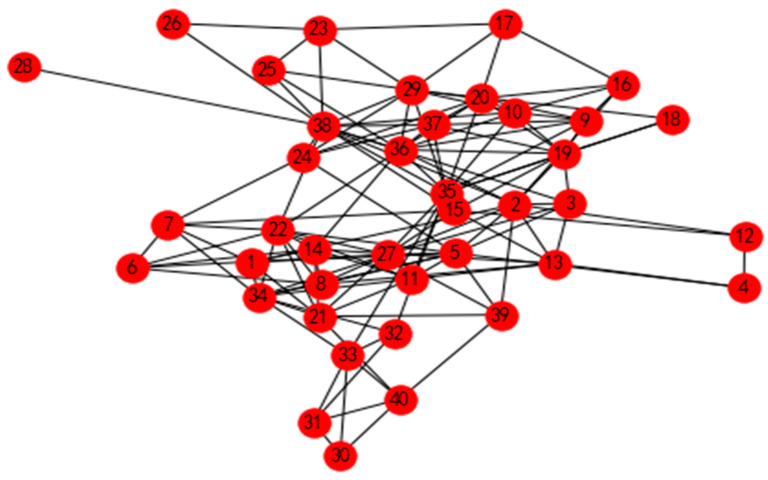
The original social relationship between decision makers without considering trust–distrust values.

**Figure 9:**
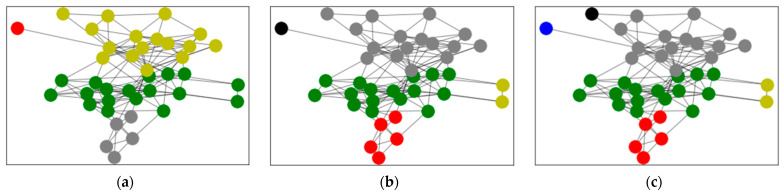
The aggregation result without considering trust relations between decision makers. (**a**) shows the aggregation result of 4 subgroups; (**b**) shows the aggregation result of 5 subgroups; (**c**) shows the aggregation result of subgroups.

To evaluate the novel method proposed in this paper, we compared our results to those of the method in reference [29], which is a cluster analysis that does not consider the trust relationship between decision makers. The original social relationship of decision makers is shown in Figure 8. We implemented the aggregating method of reference [29], and the clustering result is displayed in Figure 9. The results of this method led to separate decision-making in each cluster. This shows that the methods fail to consider the trust relationships among decision makers, which impacts the results of the clustering method.

**Figure 10:**
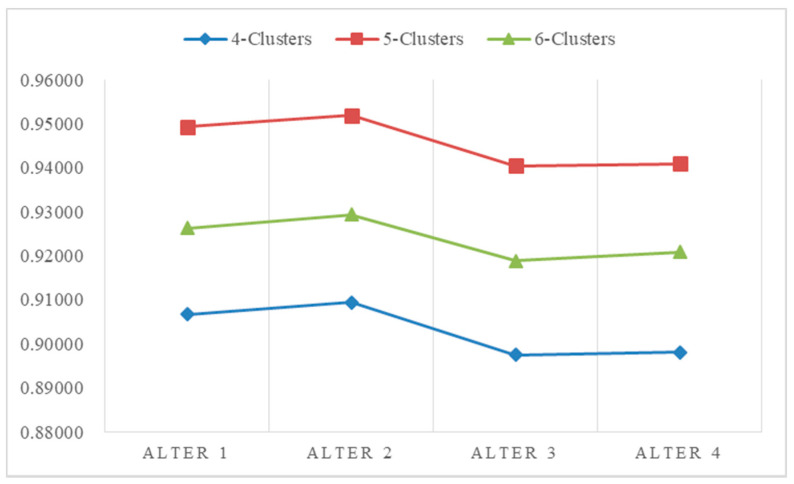
Ranking results without considering decision makers’ trust relations.

The aggregated results of 40 decision makers can be found in Table 4, where numbers are the order of decision makers. The ranking results of different numbers of clusters are shown in Table 5 and Figure 9. Figure 9a shows the aggregation result of four subgroups, where the green points stand for Group A, the yellow points stand for Group B, the grey points stand for Group C and the red points stand for Group D. Figure 9b shows the aggregation result of five subgroups, where the green points stand for Group A, the yellow points stand for Group B, the grey points stand for Group C, the red points stand for Group D and the black points stand for Group E. Figure 9c shows the aggregation result of six subgroups, where the green points stand for Group A, the yellow points stand for Group B, the grey points stand for Group C, the red points stand for Group D, the blue points stand for Group E and the yellow points stand for Group F. The ranking results without considering decision makers trust and distrust relations, which could be found in Figure 10 directly. 

The results in Table 6 show that the best alternative is the same as that shown in Table 4. Overall, the modularity is worse than considering the trust relationship between decision makers. The best modularity without considering the trust relationship is 0.4126, and the best while considering the trust relationship is 0.4150. In addition, the aggregated results of considering trust relations are better, as they do not have a single decision maker making up a subgroup. Generally, the best alternative is the same whether considering the trust relations or not, while the concrete ranking results are different comparing Table 3 and Table 5. Thus, based on Figure 10, developing eco-tourism focusing on farm specialties is the best choice for this region’s development. 

## 5. Conclusions

China has embraced environmental protection as a fundamental national policy and has implemented a strict ecological and environmental protection system in order to promote green lifestyles and models for development. At present, a conflict still exists between the interests of economic development and the environment, and the resulting economic and social problems are severe. In the era of the low-carbon economy, the two major industries of culture and tourism have gradually merged to become the “green sunrise industry” and are a priority in world development. As “pillar industries of the national economy”, the industries of culture and tourism have been increasingly integrated. The path of development of the cultural tourism industry will have a great impact on the creation of an ecological civilization. The careful analysis of cultural tourism projects plays a key role in their sustainable development. 

In this paper, we performed an in-depth analysis of the optimal decision-making for assessing rural cultural tourism projects. Our approach extends the research methods and application of fuzzy decision theory and provides a framework for the quantitative analysis of regional cultural projects by the relevant government departments. Three main points are obtained: a. the trust–distrust value is introduced to measure the decision-makers’ relationship, obtaining a directed weighted network, and the extended GN algorithm is used to cope with the computational challenges of incorporating a large amount of data; b. IVIFN is placed in the rectangular coordinate system, whose geometric significance is analyzed, and the integration operator of the initial evaluation information is calculated; c. a clustering method model is proposed which is suitable for LSGDM in a fuzzy environment. The significant contribution of this paper is calculating the trust degree and distrust degree between different decision makers because people’s trust and distrust relationships are obviously different with each other. Most of the existing papers only focus on the existence of social network relations, while the proposed algorithm in this paper focuses on the directivity and intensity of network relations between decision makers. The data in Appendix C show that the trust–distrust value between decision makers is different and has a certain influence on the clustering result. Based on the results of our research, optimal analysis of cultural tourism projects should consider input from the government, participating businesses and all sectors of society in order to achieve market-oriented operations and sustainable development. This paper evaluated the development potential of cultural tourism in a township in Hebei Province, China, and determined the most suitable development model for cultural tourism. Finally, the best decision-making framework was determined for assessing future rural cultural tourism projects. In future research, on the one hand, we will focus on the measure of calculating trust–distrust relationship degree, and on the other hand, the aggregating algorithm including a weighted directed network should also be improved considering the decision makers’ communication issue. These two points really exist in reality and have an important influence on the final evaluation result.

## Figures and Tables

**Table 1:** Example of a trust–distrust values.

Num-DM	Num-DM	(*t*_12_, *d*_12_)	(*t*_21_, *d*_21_)	Average Trust Degree	Average Accuracy Degree
1	2	(0.55, 0.35)	(0,0)	0.1	0.45

**Table 2:** Comparison results for different distance measures.

Method	*D*_1_(*P*_1_, *Q*)	*D*_2_(*P*_1_, *Q*)	Comparison	Classing Result
Reference [54]	0.13	0.13	*D*_1_(*P*_1_, *Q*) = *D*_2_(*P*_2_, *Q*)	*Q* could not be determined.
Reference [55]	0.28	0.24	*D*_1_(*P*_1_, *Q*) ≥ *D*_2_(*P*_2_, *Q*)	*Q* should be classified into team *P*_2_.
Reference [56]	1.38	1.30	*D*_1_(*P*_1_, *Q*) ≥ *D*_2_(*P*_2,_ *Q*)	*Q* should be classified into team *P*_2_.
This paper	2.16	2.15	*D*_1_(*P*_1_, *Q*) ≥ *D*_2_(*P*_2_, *Q*)	*Q* should be classified into team *P*_2_.

**Table 3:** Aggregating result of decision makers considering trust relations.

Aggregating Result	Decision Makers of Each Subgroup
4-Clusters	A-[‘10’, ‘16’, ‘17’, ‘18’, ‘19’, ‘20’, ‘23’, ‘24’, ‘25’, ‘26’, ‘28’, ‘29’, ‘35’, ‘36’, ‘37’, ‘38’, ‘9’] B-[‘1’, ‘11’, ‘14’, ‘2’, ‘21’, ‘22’, ‘27’, ‘3’, ‘32’, ‘34’, ‘39’, ‘5’, ‘6’, ‘7’, ‘8’] C-[‘12’, ‘13’, ‘15’, ‘4’] D-[‘30’, ‘31’, ‘33’, ‘40’]
5-Clusters	A-[‘10’, ‘16’, ‘17’, ‘18’, ‘19’, ‘20’, ‘23’, ‘24’, ‘25’, ‘26’, ‘28’, ‘29’, ‘35’, ‘36’, ‘37’, ‘38’, ‘9’] B-[‘1’, ‘11’, ‘14’, ‘21’, ‘22’, ‘27’, ‘32’, ‘34’, ‘39’, ‘5’, ‘6’, ‘7’, ‘8’] C-[‘12’, ‘13’, ‘15’, ‘4’] D-[‘30’, ‘31’, ‘33’, ‘40’] E-[‘2’, ‘3’]
6-Clusters	A-[‘1’, ‘11’, ‘14’, ‘21’, ‘22’, ‘27’, ‘32’, ‘34’, ‘39’, ‘5’, ‘6’, ‘7’, ‘8’] B-[‘10’, ‘16’, ‘17’, ‘18’, ‘19’, ‘20’, ‘24’, ‘25’, ‘29’, ‘35’, ‘36’, ‘37’, ‘9’] C-[‘12’, ‘13’, ‘15’, ‘4’] D-[‘23’, ‘26’, ‘28’, ‘38’] E-[‘30’, ‘31’, ‘33’, ‘40’] F-[‘2’, ‘3’]

**Table 4:** Aggregating results and modularity considering trust–distrust relations.

Aggregating Result		4-Clusters	5-Clusters	6-Clusters
Modularity		0.4141	0.4150	0.4034
Ranking result	1st	$\mathrm{Alter}\;A_{2}$	$\mathrm{Alter}\;A_{2}$	$\mathrm{Alter}\;A_{2}$
	2nd	$\mathrm{Alter}\;A_{3}$	$\mathrm{Alter}\;A_{1}$	$\mathrm{Alter}\;A_{1}$
	3rd	$\mathrm{Alter}\;A_{1}$	$\mathrm{Alter}\;A_{4}$	$\mathrm{Alter}\;A_{4}$
	4th	$\mathrm{Alter}\;A_{4}$	$\mathrm{Alter}\;A_{3}$	$\mathrm{Alter}\;A_{3}$

**Table 5:** Aggregating result of decision makers without considering trust relations.

Aggregating Result	Decision Makers of Each Subgroup
4-Clusters	A-[‘1’, ‘11’, ‘12’, ‘13’, ‘14’, ‘15’, ‘2’, ‘21’, ‘22’, ‘27’, ‘3’, ‘34’, ‘39’, ‘4’, ‘5’, ‘6’, ‘7’, ‘8’] B-[‘10’, ‘16’, ‘17’, ‘18’, ‘19’, ‘20’, ‘23’, ‘24’, ‘25’, ‘26’, ‘29’, ‘35’, ‘36’, ‘37’, ‘38’, ‘9’] B-[‘30’, ‘31’, ‘32’, ‘33’, ‘40’] D-[‘28’]
5-Clusters	A-[‘1’, ‘11’, ‘13’, ‘14’, ‘15’, ‘2’, ‘21’, ‘22’, ‘27’, ‘3’, ‘34’, ‘39’, ‘5’, ‘6’, ‘7’, ‘8’] B-[‘10’, ‘16’, ‘17’, ‘18’, ‘19’, ‘20’, ‘23’, ‘24’, ‘25’, ‘26’, ‘29’, ‘35’, ‘36’, ‘37’, ‘38’, ‘9’] C-[‘30’, ‘31’, ‘32’, ‘33’, ‘40’] D-[‘12’, ‘4’] E-[‘28’]
6-Clusters	A-[‘1’, ‘11’, ‘13’, ‘14’, ‘15’, ‘2’, ‘21’, ‘22’, ‘27’, ‘3’, ‘34’, ‘39’, ‘5’, ‘6’, ‘7’, ‘8’, ‘15’] B-[‘12’, ‘4’] C-[‘10’, ‘16’, ‘17’, ‘18’, ‘19’, ‘20’, ‘23’, ‘24’, ‘25’, ‘29’, ‘35’, ‘36’, ‘37’, ‘38’, ‘9’] D-[‘30’, ‘31’, ‘32’, ‘33’, ‘40’] E-[‘26’], F-[‘28’]

**Table 6:** Aggregating results and modularity without considering trust relations.

Aggregating Result		4-Clusters	5-Clusters	6-Clusters
Modularity		0.4126	0.4034	0.3950
Ranking result	1st	Alter	$\mathrm{Alter}\;A_{2}$	$\mathrm{Alter}\;A_{2}$
	2ed	$\mathrm{Alter}\;A_{1}$	$\mathrm{Alter}\;A_{1}$	$\mathrm{Alter}\;A_{1}$
	3rd	$\mathrm{Alter}\;A_{4}$	$\mathrm{Alter}\;A_{4}$	$\mathrm{Alter}\;A_{4}$
	4th	$\mathrm{Alter}\;A_{3}$	$\mathrm{Alter}\;A_{3}$	$\mathrm{Alter}\;A_{3}$

## Data Availability

The data presented in this study are available on request from the Appendix.

## Appendix A

**Table A1:** Trust-distrust relationships between 40 decision makers.

DM(A)	DM(B)	(*t*_AB_, *d*_AB_)	(*t*_BA_, *d*_BA_)	Average Trust Degree	Average Accuracy Degree
1	2	(0.55, 0.35)	(0,0)	0.1	0.45
1	5	(0.45, 0.4)	(0.45, 0.35)	0.075	0.825
1	7	(0.55, 0.35)	(0.65, 0.45)	0.2	1
1	14	(0.3, 0.4)	(0.35, 0.45)	0	0.75
1	21	(0.65, 0.15)	(0.6, 0.1)	0.5	0.75
1	34	(0.55, 0.65)	(0.6, 0.2)	0.15	1
2	3	(0.2, 0.1)	(0.45, 0.1)	0.225	0.425
2	5	(0.25, 0.3)	(0.4, 0.15)	0.1	0.55
2	12	(0.35, 0.4)	(0.35, 0.3)	0	0.7
2	13	(0.35, 0.25)	(0.45, 0.4)	0.075	0.725
2	16	(0.2, 0.25)	(0.2, 0.1)	0.025	0.375
2	19	(0.45, 0.4)	(0.55, 0.5)	0.05	0.95
2	27	(0.3, 0.35)	(0.45, 0.55)	0	0.825
2	36	(0.35, 0.4)	(0.6, 0.2)	0.175	0.775
2	38	(0.25, 0.2)	(0.45, 0.45)	0.025	0.675
2	39	(0.45, 0.5)	(0.45, 0.45)	0	0.925
3	5	(0.55, 0.6)	(0.8, 0.7)	0.025	1.325
3	13	(0.35, 0.6)	(0.3, 0.4)	0	0.825
3	19	(0.45, 0.5)	(0.3, 0.35)	0	0.8
3	27	(0.65, 0.2)	(0.25, 0.3)	0.2	0.7
3	38	(0.45, 0.4)	(0.35, 0.3)	0.05	0.75
4	5	(0.5, 0.45)	(0.35, 0.3)	0.05	0.8
4	12	(0.55, 0.6)	(0.15, 0.2)	0	0.75
4	13	(0.6, 0.2)	(0.15, 0.3)	0.125	0.625
5	8	(0.7, 0.65)	(0.35, 0.3)	0.05	1
5	11	(0.25, 0.2)	(0.65, 0.6)	0.05	0.85
5	22	(0.3, 0.4)	(0.35, 0.2)	0.025	0.625
5	24	(0.45, 0.2)	(0.45, 0.2)	0.25	0.65
5	27	(0.75, 0.25)	(0.2, 0.1)	0.3	0.65
5	34	(0.7, 0.2)	(0.65, 0.25)	0.45	0.9
5	39	(0.55, 0.5)	(0.45, 0.4)	0.05	0.95
6	7	(0.45, 0.2)	(0.4, 0.15)	0.25	0.6
6	8	(0.45, 0.1)	(0.45, 0.05)	0.375	0.525
6	14	(0.7, 0.45)	(0.7, 0.1)	0.425	0.975
6	22	(0.6, 0.25)	(0.65, 0.1)	0.45	0.8
6	27	(0.65, 0.25)	(0.6, 0.3)	0.35	0.9
7	22	(0.25, 0.1)	(0.3, 0.15)	0.15	0.4
7	24	(0.3, 0.15)	(0.35, 0.2)	0.15	0.5
7	34	(0.35, 0.2)	(0.25, 0.1)	0.15	0.45
8	13	(0.7, 0.2)	(0.65, 0.25)	0.45	0.9
8	14	(0.55, 0.5)	(0.45, 0.4)	0.05	0.95
8	22	(0.45, 0.2)	(0.4, 0.15)	0.25	0.6
8	27	(0.8, 0.1)	(0.55, 0.2)	0.525	0.825
8	34	(0.45, 0.1)	(0.45, 0.05)	0.375	0.525
9	10	(0.7, 0.45)	(0.7, 0.1)	0.425	0.975
9	16	(0.6, 0.25)	(0.65, 0.1)	0.45	0.8
9	19	(0.65, 0.25)	(0.6, 0.3)	0.35	0.9
9	20	(0.25, 0.1)	(0.3, 0.15)	0.15	0.4
9	35	(0.7, 0.8)	(0.3, 0.25)	0	1.025
9	36	(0.65, 0.25)	(0.6, 0.3)	0.35	0.9
9	37	(0.25, 0.1)	(0.3, 0.15)	0.15	0.4
10	16	(0.7, 0.2)	(0.65, 0.25)	0.45	0.9
10	19	(0.55, 0.5)	(0.45, 0.4)	0.05	0.95
10	20	(0.45, 0.2)	(0.4, 0.15)	0.25	0.6
10	29	(0.8, 0.1)	(0.55, 0.2)	0.525	0.825
10	35	(0.65, 0.2)	(0.25, 0.1)	0.3	0.6
10	36	(0.6, 0.25)	(0.65, 0.1)	0.45	0.8
10	38	(0.65, 0.25)	(0.6, 0.3)	0.35	0.9
11	13	(0.25, 0.1)	(0.3, 0.15)	0.15	0.4
11	14	(0.7, 0.8)	(0.3, 0.25)	0	1.025
11	21	(0.65, 0.25)	(0.6, 0.3)	0.35	0.9
11	22	(0.25, 0.1)	(0.3, 0.15)	0.15	0.4
11	34	(0.65, 0.25)	(0.6, 0.3)	0.35	0.9
11	35	(0.85, 0.25)	(0.15, 0.1)	0.325	0.675
13	14	(0.75, 0.25)	(0.2, 0.1)	0.3	0.65
13	15	(0.7, 0.2)	(0.65, 0.25)	0.45	0.9
14	21	(0.55, 0.5)	(0.45, 0.4)	0.05	0.95
14	22	(0.45, 0.2)	(0.4, 0.15)	0.25	0.6
14	36	(0.45, 0.1)	(0.45, 0.05)	0.375	0.525
15	7	(0.35, 0.4)	(0.35, 0.3)	0	0.7
15	12	(0.35, 0.25)	(0.45, 0.4)	0.075	0.725
15	19	(0.2, 0.25)	(0.2, 0.1)	0.025	0.375
15	27	(0.45, 0.4)	(0.55, 0.5)	0.05	0.95
15	38	(0.3, 0.35)	(0.45, 0.55)	0	0.825
16	17	(0.7, 0.2)	(0.65, 0.25)	0.45	0.9
16	20	(0.55, 0.5)	(0.45, 0.4)	0.05	0.95
17	20	(0.45, 0.2)	(0.4, 0.15)	0.25	0.6
17	23	(0.8, 0.1)	(0.55, 0.2)	0.525	0.825
17	29	(0.65, 0.2)	(0.25, 0.1)	0.3	0.6
18	19	(0.6, 0.25)	(0.65, 0.1)	0.45	0.8
18	29	(0.6, 0.25)	(0.65, 0.1)	0.45	0.8
18	35	(0.55, 0.2)	(0.45, 0.2)	0.3	0.7
19	20	(0.7, 0.2)	(0.65, 0.25)	0.45	0.9
19	35	(0.55, 0.5)	(0.45, 0.4)	0.05	0.95
19	36	(0.45, 0.2)	(0.4, 0.15)	0.25	0.6
19	37	(0.8, 0.1)	(0.55, 0.2)	0.525	0.825
20	24	(0.65, 0.2)	(0.25, 0.1)	0.3	0.6
20	29	(0.6, 0.25)	(0.65, 0.1)	0.45	0.8
20	35	(0.65, 0.25)	(0.45, 0.3)	0.275	0.825
20	36	(0.45, 0.2)	(0.4, 0.15)	0.25	0.6
20	37	(0.8, 0.1)	(0.55, 0.2)	0.525	0.825
21	22	(0.65, 0.2)	(0.25, 0.1)	0.3	0.6
21	34	(0.6, 0.25)	(0.65, 0.1)	0.45	0.8
21	39	(0.65, 0.25)	(0.6, 0.3)	0.35	0.9
21	40	(0.7, 0.65)	(0.35, 0.3)	0.05	1
21	27	(0.85, 0.2)	(0.25, 0.1)	0.4	0.7
22	34	(0.25, 0.2)	(0.65, 0.6)	0.05	0.85
22	36	(0.3, 0.4)	(0.35, 0.2)	0.025	0.625
22	38	(0.45, 0.2)	(0.45, 0.2)	0.25	0.65
22	27	(0.8, 0.2)	(0.25, 0.1)	0.375	0.675
23	25	(0.6, 0.25)	(0.65, 0.1)	0.45	0.8
23	29	(0.6, 0.25)	(0.65, 0.1)	0.45	0.8
23	26	(0.55, 0.2)	(0.45, 0.2)	0.3	0.7
23	38	(0.7, 0.2)	(0.65, 0.25)	0.45	0.9
24	29	(0.55, 0.5)	(0.45, 0.4)	0.05	0.95
24	37	(0.45, 0.2)	(0.4, 0.15)	0.25	0.6
24	38	(0.8, 0.1)	(0.55, 0.2)	0.525	0.825
25	29	(0.65, 0.2)	(0.25, 0.1)	0.3	0.6
25	36	(0.6, 0.25)	(0.65, 0.1)	0.45	0.8
25	38	(0.65, 0.25)	(0.45, 0.3)	0.275	0.825
26	38	(0.45, 0.2)	(0.4, 0.15)	0.25	0.6
27	34	(0.2, 0.25)	(0.75, 0.1)	0.3	0.65
27	35	(0.55, 0.6)	(0.8, 0.1)	0.325	1.025
27	39	(0.7, 0.55)	(0.35, 0.1)	0.2	0.85
28	38	(0.75, 0.25)	(0.2, 0.1)	0.3	0.65
29	35	(0.2, 0.05)	(0.45, 0.5)	0.05	0.6
29	36	(0.7, 0.2)	(0.65, 0.25)	0.45	0.9
29	38	(0.55, 0.5)	(0.45, 0.4)	0.05	0.95
30	31	(0.45, 0.4)	(0.35, 0.3)	0.05	0.75
30	33	(0.5, 0.45)	(0.35, 0.3)	0.05	0.8
30	40	(0.55, 0.6)	(0.15, 0.2)	0	0.75
31	32	(0.6, 0.2)	(0.15, 0.3)	0.125	0.625
31	33	(0.7, 0.65)	(0.35, 0.3)	0.05	1
31	40	(0.25, 0.2)	(0.65, 0.6)	0.05	0.85
32	33	(0.3, 0.4)	(0.35, 0.2)	0.025	0.625
32	34	(0.45, 0.2)	(0.45, 0.2)	0.25	0.65
32	35	(0.2, 0.05)	(0.6, 0.2)	0.275	0.525
33	34	(0.5, 0.1)	(0.5, 0.15)	0.375	0.625
33	35	(0.4, 0.2)	(0.5, 0.45)	0.125	0.775
33	40	(0.45, 0.1)	(0.5, 0.15)	0.35	0.6
35	36	(0.25, 0.3)	(0.45, 0.2)	0.1	0.6
35	37	(0.3, 0.1)	(0.65, 0.2)	0.325	0.625
35	38	(0.35, 0.4)	(0.65, 0.1)	0.25	0.75
36	37	(0.75, 0.1)	(0.2, 0.15)	0.35	0.6
36	38	(0.7, 0.2)	(0.15, 0.2)	0.225	0.625
37	38	(0.55, 0.6)	(0.25, 0.15)	0.025	0.775
39	40	(0.2, 0.15)	(0.1, 0.05)	0.05	0.25

## Appendix B

### Appendix B.1. Research Status Analysis on Cultural Tourism

Since the 1980s, cultural tourism has grown in the international tourism market and has gradually become one of the cornerstones of modern tourism. In the current developmental atmosphere in China, the cultural tourism industry has emerged and has presented good prospects for development. Based on industrial integration theory and the standard of industrial formation, cultural tourism is in its early stages in China. Applying optimal decision-making to the assessment of cultural tourism projects is key to the success of the development of the cultural tourism industry. China’s Ministry of Culture and Ministry of Tourism Administration merged to create the Ministry of Culture and Tourism in 2018, indicating a close relationship between the development of culture and tourism. When implementing cultural tourism projects, we must keep in mind the local cultural characteristics and the needs of the public.

#### Appendix B.1.1. Study on the Tourism Development of China

A study found that both the available tourist services and the attractiveness of the destination positively affect the prospects for tourism; this study showed that the economic and social interests of tourism are not in conflict but can be mutually beneficial [65]. At present, there are two key perspectives in the research regarding tourism projects. First, studies focusing on the customer perception of destinations have been conducted to assess the factors influencing tourist perceptions [66]. Factor analysis [67] was employed to verify the impact of tourists’ perceived state in different aspects of their travel behavior by building structural equations, using GIS and other methods to analyze the rational development planning of forest resources in Beijing, Tianjin and Hebei [68]. Second, studies of the factors influencing tourist behavior and their subjective perceptions demonstrated that positive views of local culture by tourists had a positive impact on tourism behavior. A thorough understanding of what motivates tourists to select their destinations could help in offering targeted products and services. As a result, tourists will have more positive experiences and fewer negative feelings, which in turn makes them more likely to ignore negative information [69]. With the advent of the “two micro” life era in China, microblogs and WeChat (similar to Facebook and Twitter) have become popular means by which many people obtain information regarding tourist destinations. Based on the rise and development of the internet, the social network relationship between decision makers cannot be ignored when looking for solutions to complex problems. Therefore, in this paper, we constructed a weighted directed network considering the trust relationship between decision makers to improve the evaluation of potential projects in tourism.

#### Appendix B.1.2. Cultural-Based Tourism Projects

With the rise of the experience economy, tourists are attributing importance to spiritual enjoyment, and the demand for cultural experiences in tourism is increasing every day. It is important to find the best pathway for the realization of the market value of cultural products. Because culture is playing an increasingly important role in economic development, more attention has been given to this important feature of the tourism industry [70]. Culture has a significant and profound effect on economic development, and, in turn, the evolution of culture is also being influenced by economic development. As a “smoke-free industry” and “sunrise industry”, tourism has become an important path for the industrial transformation of Shanxi in the current period [71]. The study of trends in tourism should continue to be advanced at the levels of theory, methodology and application and specifically regarding the culture of village tourism [72]. Developments in the fields of culture and art are precious resources that can stimulate the vitality of the tourism industry and, thereby, become a bright spot of economic growth. Taking the development of the Wild Sanpo in Hebei Province as an example, it is apparent that the development of tourism has had structural impacts on rural life, production and ecology, and the growth of the industry needs to be accompanied by a reasonable system to ensure the sustainable development of the environment and local culture.

Historical towns with unique histories have become increasingly important tourist attractions in China, and the development of historical town tourism will inevitably have an impact on the residents’ quality of life. Taking the ancient city of Phoenix as an example, it was found that respondents had different perceptions of the impact of tourism. The material and nonmaterial quality of life of the residents of historical towns has a significantly positive impact on their overall quality of life [73]. In this era of globalization, the mismatch between the supply and demand of tourist destinations is becoming increasingly apparent. Case study data has showed that tourists attach the highest importance to history and culture, travel accommodations and convenience, and preserving the environment [39]. Important changes have taken place in the land use, employment model and industrial structure of traditional villages, and the development of tourism has become an important driving factor in the urbanization of traditional villages [74].

The main modes for the development of the cultural tourism industry locally and abroad are government guidance, market development and the combination of government and market. International cultural tourism typically is driven by the combination of government and market. The market system is relatively complete, its relevant laws and regulations are sound, and the positioning of government functions is relatively reasonable. The market plays a leading role in the development of cultural tourism products and in regulating the allocation of industrial elements, and cultural tourism resources have been well developed and utilized. However, domestically, the development of the cultural tourism industry began relatively late, and there is a vague understanding of the government’s function and market role in the development of the cultural tourism industry. The government-led development model has been adopted by most regions of China. In terms of planning and development, marketing planning, fundraising and the daily management of cultural and tourism resources, the government handles a large number of considerations and does not distinguish between government affairs and enterprises, regardless of the costs and benefits. The management of resources in terms of development and utilization may not be sustained. The laissez-faire market development model has been adopted by some individual regions. Under the circumstances of imperfect laws and regulations and inadequate supervision, there is not only a lack of scientific guidance but also a lack of effective daily management of cultural tourism resources. How to learn from the experience and lessons of the development of the cultural tourism industry at home and abroad and how to form a regional characteristic cultural tourism industry in line with local conditions are important issues to be solved. One approach is to reasonably define the boundaries of the government’s functional authority and its role in the market, which requires a series of supporting policies and legal systems. It is important to closely integrate the regional characteristics of cultural and tourist resources in different regions, and it is necessary to fully mobilize the broad participation of the public in cultural tourism resources and protect cultural tourism resources while developing them. Based on the scientific quantitative evaluation of the value of cultural resources, it has become one of the important issues in this project to optimize the matching efficiency of regional cultural tourism resources.

## Appendix C. Construction of Evaluation Mechanism of Cultural Tourism

At present, the conflict between economic development and environmental protection still exists in some areas of China. Because of the “Horse culture and tourism economy in Anping County” and the latest statistics regarding the economic benefits of cultural tourism, it is clear that China has achieved positive results. This shows that China’s cultural tourism industry has developed while also promoting the creation of an ecological civilization to a degree. Based on the empirical results, there is a clear path forward for the development of cultural tourism projects. This paper examines the optimal decision-making paradigm for cultural tourism projects.

Our approach introduces the public into the decision-making process, thereby enriching the pool of decision makers. The number of public groups is large, and the degree of heterogeneity is high. In the age of the internet, information can be shared easily by people in different places; therefore, it is difficult to track trends in information sharing among public groups. However, we can gain an understanding of the opinions of public groups more widely and objectively by selecting public representatives. For the most part, cultural tourism projects are profitable enterprises that benefit public groups. It is important for the government and businesses to accurately grasp the key factors that influence a group’s behavior, including their educational background, age, gender, nature of work, values, income, etc.

First, we determined the trust relationship among the public groups participating in the survey. In our model, each individual is represented by a point. Trust values are assigned, and the group-directed weighted network structure is obtained. Then, the analysis dimension of the evaluation index system is determined. Based on the analysis of questionnaire responses, the evaluation is carried out based on the following four dimensions:

$C_{1}$: Natural environment dimension, including trees, vegetation, mountains and rivers;

$C_{2}$: Social environment dimension, including public security, culture, morality and history;

$C_{3}$: Economic environment dimension, including regional GDP index, infrastructure improvement level, etc.;

$C_{4}$: Stakeholder dimension, including the folk customs of residents in the region.

We applied this method to the analysis of a township in Hebei Province as an example. One feature of the region is that it has effective, convenient transportation. Fruit trees are an important resource in the region, and many farmers sell fruit for their living. However, the overall household income is low, and the revenue is highly dependent on the natural environment. Serious natural disasters have a great impact on the quality of life of farmers in the region. Therefore, the local government should develop projects in line with China’s initiative promoting cultural tourism but also keep in mind local features of the area. Based on the recommendation of experts, four cultural tourism projects have been proposed:

$A_{1}$: Restore a culturally important historical town;

$A_{2}$: Develop eco-tourism focusing on farm specialties;

$A_{3}$: Promote leisure tourism based on the weather;

$A_{4}$: Build a new fashion tourism village.

The following tables are given by the remaining 40 decision makers of the illustrative example.

**Table A2:** The decision result given by DM_1_.

	Criterion1	Criterion2	Criterion3	Criterion4	Criterion5
Alter-1	<[0.25, 0.35], [0.45, 0.55]>	<[0.55, 0.60], [0.35, 0.40]>	<[0.15, 0.30], [0.60, 0.65]>	<[0.45, 0.50], [0.40, 0.50]>	<[0.35, 0.40], [0.25, 0.35]>
Alter-2	<[0.55, 0.65], [0.10, 0.30]>	<[0.35, 0.40], [0.25, 0.30]>	<[0.40, 0.50], [0.40, 0.45]>	<[0.35, 0.50], [0.30, 0.35]>	<[0.45, 0.50], [0.20, 0.35]>
Alter-3	<[0.65, 0.75], [0.10, 0.20]>	<[0.55, 0.65], [0.20, 0.30]>	<[0.45, 0.50], [0.30, 0.40]>	<[0.45, 0.50], [0.25, 0.30]>	<[0.75, 0.80], [0.10, 0.15]>
Alter-4	<[0.45, 0.55], [0.25, 0.35]>	<[0.45, 0.50], [0.20, 0.45]>	<[0.60, 0.65], [0.25, 0.30]>	<[0.50, 0.55], [0.10, 0.35]>	<[0.40, 0.50], [0.40, 0.50]>

**Table A3:** The decision result given by DM_2_.

	Criterion1	Criterion2	Criterion3	Criterion4	Criterion5
Alter-1	<[0.45, 0.55], [0.35, 0.40]>	<[0.50, 0.60], [0.20, 0.30]>	<[0.20, 0.30], [0.50, 0.60]>	<[0.40, 0.50], [0.40, 0.50]>	<[0.65, 0.70], [0.10, 0.20]>
Alter-2	<[0.60, 0.65], [0.20, 0.30]>	<[0.65, 0.70], [0.25, 0.30]>	<[0.40, 0.50], [0.25, 0.30]>	<[0.40, 0.50], [0.30, 0.40]>	<[0.60, 0.65], [0.20, 0.30]>
Alter-3	<[0.40, 0.50], [0.30, 0.40]>	<[0.30, 0.40], [0.10, 0.20]>	<[0.30, 0.40], [0.30, 0.40]>	<[0.50, 0.60], [0.10, 0.30]>	<[0.55, 0.60], [0.20, 0.30]>
Alter-4	<[0.50, 0.60], [0.20, 0.30]>	<[0.40, 0.50], [0.20, 0.30]>	<[0.60, 0.70], [0.20, 0.30]>	<[0.40, 0.50], [0.10, 0.20]>	<[0.75, 0.80], [0.15, 0.20]>

**Table A4:** The decision result given by DM_3_.

	Criterion1	Criterion2	Criterion3	Criterion4	Criterion5
Alter-1	<[0.55, 0.65], [0.20, 0.35]>	<[0.55, 0.60], [0.10, 0.30]>	<[0.45, 0.55], [0.25, 0.35]>	<[0.55, 0.60], [0.15, 0.35]>	<[0.35, 0.45], [0.40, 0.50]>
Alter-2	<[0.70, 0.75], [0.10, 0.20]>	<[0.65, 0.75], [0.15, 0.20]>	<[0.55, 0.60], [0.35, 0.40]>	<[0.60, 0.65], [0.30, 0.35]>	<[0.40, 0.50], [0.20, 0.30]>
Alter-3	<[0.45, 0.55], [0.25, 0.35]>	<[0.65, 0.70], [0.20, 0.25]>	<[0.55, 0.65], [0.25, 0.35]>	<[0.65, 0.70], [0.2, 0.30]>	<[0.75, 0.80], [0.15, 0.20]>
Alter-4	<[0.40, 0.50], [0.40, 0.55]>	<[0.60, 0.65], [0.10, 0.15]>	<[0.45, 0.50], [0.35, 0.45]>	<[0.65, 0.70], [0.25, 0.30]>	<[0.65, 0.70], [0.15, 0.20]>

**Table A5:** The decision result given by DM_4_.

	Criterion1	Criterion2	Criterion3	Criterion4	Criterion5
Alter-1	<[0.50, 0.55], [0.30, 0.40]>	<[0.50, 0.55], [0.20, 0.30]>	<[0.40, 0.45], [0.20, 0.25]>	<[0.50, 0.55], [0.25, 0.35]>	<[0.65, 0.70], [0.20, 0.25]>
Alter-2	<[0.75, 0.80], [0.05, 0.10]>	<[0.35, 0.45], [0.45, 0.50]>	<[0.75, 0.80], [0.15, 0.20]>	<[0.80, 0.85], [0.10, 0.15]>	<[0.55, 0.65], [0.30, 0.35]>
Alter-3	<[0.65, 0.70], [0.15, 0.20]>	<[0.55, 0.60], [0.35, 0.40]>	<[0.55, 0.60], [0.20, 0.25]>	<[0.65, 0.70], [0.15, 0.20]>	<[0.70, 0.75], [0.20, 0.25]>
Alter-4	<[0.60, 0.65], [0.25, 0.30]>	<[0.50, 0.55], [0.25, 0.30]>	<[0.50, 0.55], [0.15, 0.20]>	<[0.55, 0.60], [0.25, 0.30]>	<[0.60, 0.65], [0.25, 0.30]>

**Table A6:** The decision result given by DM_5_.

	Criterion1	Criterion2	Criterion3	Criterion4	Criterion5
Alter-1	<[0.65, 0.70], [0.20, 0.25]>	<[0.75, 0.85], [0.10, 0.15]>	<[0.60, 0.65], [0.20, 0.25]>	<[0.75, 0.80], [0.10, 0.15]>	<[0.80, 0.85], [0.10, 0.15]>
Alter-2	<[0.60, 0.70], [0.30, 0.35]>	<[0.60, 0.70], [0.15, 0.20]>	<[0.65, 0.70], [0.20, 0.25]>	<[0.70, 0.75], [0.20, 0.25]>	<[0.60, 0.65], [0.25, 0.30]>
Alter-3	<[0.80, 0.85], [0.05, 0.10]>	<[0.80, 0.85], [0.10, 0.15]>	<[0.75, 0.80], [0.15, 0.20]>	<[0.85, 0.95], [0.00, 0.05]>	<[0.70, 0.75], [0.20, 0.25]>
Alter-4	<[0.55, 0.60], [0.25, 0.35]>	<[0.60, 0.65], [0.25, 0.30]>	<[0.55, 0.60], [0.25, 0.30]>	<[0.60, 0.65], [0.25, 0.30]>	<[0.55, 0.60], [0.30, 0.35]>

**Table A7:** The decision result given by DM_6_.

	Criterion1	Criterion2	Criterion3	Criterion4	Criterion5
Alter-1	<[0.65, 0.70], [0.25, 0.30]>	<[0.60, 0.65], [0.25, 0.30]>	<[0.75, 0.80], [0.15, 0.20]>	<[0.75, 0.80], [0.10, 0.15]>	<[0.75, 0.80], [0.05, 0.10]>
Alter-2	<[0.60, 0.65], [0.25, 0.30]>	<[0.55, 0.65], [0.25, 0.20]>	<[0.65, 0.70], [0.20, 0.25]>	<[0.70, 0.75], [0.15, 0.20]>	<[0.65, 0.70], [0.15, 0.25]>
Alter-3	<[0.70, 0.75], [0.10, 0.15]>	<[0.65, 0.70], [0.20, 0.25]>	<[0.85, 0.90], [0.05, 0.10]>	<[0.85, 0.90], [0.05, 0.10]>	<[0.80, 0.85], [0.10, 0.20]>
Alter-4	<[0.55, 0.60], [0.35, 0.40]>	<[0.50, 0.55], [0.40, 0.45]>	<[0.55, 0.60], [0.30, 0.35]>	<[0.55, 0.60], [0.25, 0.30]>	<[0.60, 0.65], [0.20, 0.25]>

**Table A8:** The decision result given by DM_7_.

	Criterion1	Criterion2	Criterion3	Criterion4	Criterion5
Alter-1	<[0.70, 0.75], [0.20, 0.25]>	<[0.65, 0.70], [0.25, 0.30]>	<[0.85, 0.90], [0.00, 0.10]>	<[0.80, 0.85], [0.05, 0.15]>	<[0.75, 0.80], [0.10, 0.20]>
Alter-2	<[0.65, 0.70], [0.25, 0.30]>	<[0.60, 0.65], [0.30, 0.35]>	<[0.65, 0.70], [0.25, 0.30]>	<[0.70, 0.75], [0.20, 0.25]>	<[0.60, 0.70], [0.25, 0.30]>
Alter-3	<[0.80, 0.85], [0.10, 0.15]>	<[0.75, 0.80], [0.10, 0.15]>	<[0.90, 0.95], [0.00, 0.05]>	<[0.90, 0.95], [0.00, 0.05]>	<[0.70, 0.75], [0.20, 0.25]>
Alter-4	<[0.60, 0.65], [0.30, 0.35]>	<[0.55, 0.60], [0.25, 0.35]>	<[0.55, 0.60], [0.30, 0.35]>	<[0.65, 0.70], [0.25, 0.30]>	<[0.55, 0.65], [0.25, 0.35]>

**Table A9:** The decision result given by DM_8_.

	Criterion1	Criterion2	Criterion3	Criterion4	Criterion5
Alter-1	<[0.75, 0.80], [0.15, 0.20]>	<[0.70, 0.75], [0.25, 0.20]>	<[0.75, 0.80], [0.10, 0.20]>	<[0.75, 0.80], [0.15, 0.20]>	<[0.85, 0.90], [0.05, 0.10]>
Alter-2	<[0.65, 0.70], [0.25, 0.30]>	<[0.65, 0.70], [0.25, 0.30]>	<[0.70, 0.75], [0.20, 0.25]>	<[0.65, 0.70], [0.20, 0.25]>	<[0.65, 0.70], [0.20, 0.25]>
Alter-3	<[0.85, 0.90], [0.05, 0.10]>	<[0.85, 0.90], [0.00, 0.05]>	<[0.80, 0.85], [0.05, 0.10]>	<[0.90, 0.95], [0.00, 0.05]>	<[0.80, 0.85], [0.10, 0.15]>
Alter-4	<[0.55, 0.60], [0.30, 0.35]>	<[0.55, 0.65], [0.30, 0.35]>	<[0.65, 0.70], [0.20, 0.25]>	<[0.60, 0.65], [0.25, 0.30]>	<[0.60, 0.65], [0.30, 0.35]>

**Table A10:** The decision result given by DM_9_.

	Criterion1	Criterion2	Criterion3	Criterion4	Criterion5
Alter-1	<[0.75, 0.80], [0.10, 0.20]>	<[0.60, 0.65], [0.30, 0.35]>	<[0.80, 0.90], [0.05, 0.10]>	<[0.65, 0.70], [0.20, 0.25]>	<[0.75, 0.85], [0.10, 0.15]>
Alter-2	<[0.60, 0.65], [0.30, 0.35]>	<[0.65, 0.70], [0.20, 0.25]>	<[0.75, 0.80], [0.15, 0.20]>	<[0.60, 0.65], [0.20, 0.25]>	<[0.70, 0.70], [0.20, 0.25]>
Alter-3	<[0.55, 0.60], [0.30, 0.40]>	<[0.55, 0.60], [0.30, 0.35]>	<[0.70, 0.75], [0.20, 0.25]>	<[0.65, 0.70], [0.15, 0.25]>	<[0.65, 0.80], [0.15, 0.20]>
Alter-4	<[0.85, 0.90], [0.05, 0.10]>	<[0.70, 0.80], [0.10, 0.15]>	<[0.90, 0.95], [0.00, 0.05]>	<[0.70, 0.75], [0.10, 0.15]>	<[0.80, 0.80], [0.05, 0.15]>

**Table A11:** The decision result given by DM_10_.

	Criterion1	Criterion2	Criterion3	Criterion4	Criterion5
Alter-1	<[0.70, 0.80], [0.15, 0.20]>	<[0.60, 0.65], [0.30, 0.35]>	<[0.75, 0.80], [0.10, 0.15]>	<[0.65, 0.70], [0.15, 0.25]>	<[0.70, 0.75], [0.15, 0.20]>
Alter-2	<[0.55, 0.65], [0.30, 0.35]>	<[0.65, 0.70], [0.15, 0.25]>	<[0.70, 0.80], [0.15, 0.20]>	<[0.60, 0.65], [0.15, 0.20]>	<[0.65, 0.70], [0.20, 0.25]>
Alter-3	<[0.65, 0.70], [0.20, 0.25]>	<[0.65, 0.75], [0.15, 0.20]>	<[0.65, 0.70], [0.15, 0.20]>	<[0.60, 0.70], [0.10, 0.25]>	<[0.70, 0.75], [0.20, 0.25]>
Alter-4	<[0.80, 0.90], [0.05, 0.10]>	<[0.75, 0.80], [0.05, 0.10]>	<[0.85, 0.90], [0.10, 0.15]>	<[0.70, 0.80], [0.15, 0.20]>	<[0.85, 0.90], [0.05, 0.10]>

**Table A12:** The decision result given by DM_11_.

	Criterion1	Criterion2	Criterion3	Criterion4	Criterion5
Alter-1	<[0.70, 0.75], [0.20, 0.25]>	<[0.60, 0.65], [0.30, 0.35]>	<[0.70, 0.75], [0.20, 0.25]>	<[0.65, 0.70], [0.20, 0.25]>	<[0.55, 0.60], [0.20, 0.25]>
Alter-2	<[0.75, 0.80], [0.15, 0.20]>	<[0.70, 0.75], [0.20, 0.25]>	<[0.80, 0.85], [0.10, 0.15]>	<[0.75, 0.80], [0.15, 0.20]>	<[0.60, 0.65], [0.30, 0.35]>
Alter-3	<[0.85, 0.90], [0.05, 0.10]>	<[0.65, 0.70], [0.25, 0.30]>	<[0.75, 0.80], [0.15, 0.20]>	<[0.70, 0.75], [0.05, 0.10]>	<[0.65, 0.70], [0.20, 0.25]>
Alter-4	<[0.65, 0.70], [0.20, 0.25]>	<[0.55, 0.60], [0.25, 0.30]>	<[0.65, 0.70], [0.15, 0.20]>	<[0.60, 0.65], [0.30, 0.35]>	<[0.50, 0.55], [0.35, 0.40]>

**Table A13:** The decision result given by DM_12_.

	Criterion1	Criterion2	Criterion3	Criterion4	Criterion5
Alter-1	<[0.70, 0.80], [0.10, 0.20]>	<[0.75, 0.80], [0.15, 0.20]>	<[0.75, 0.80], [0.10, 0.15]>	<[0.70, 0.75], [0.15, 0.25]>	<[0.70, 0.80], [0.10, 0.15]>
Alter-2	<[0.65, 0.70], [0.20, 0.25]>	<[0.65, 0.70], [0.25, 0.30]>	<[0.65, 0.70], [0.25, 0.30]>	<[0.65, 0.70], [0.20, 0.25]>	<[0.65, 0.70], [0.20, 0.25]>
Alter-3	<[0.85, 0.90], [0.05, 0.10]>	<[0.80, 0.85], [0.10, 0.20]>	<[0.80, 0.85], [0.05, 0.10]>	<[0.80, 0.85], [0.10, 0.15]>	<[0.75, 0.80], [0.05, 0.15]>
Alter-4	<[0.60, 0.65], [0.25, 0.35]>	<[0.55, 0.60], [0.30, 0.35]>	<[0.60, 0.65], [0.25, 0.35]>	<[0.60, 0.65], [0.25, 0.30]>	<[0.60, 0.65], [0.30, 0.35]>

**Table A14:** The decision result given by DM_13_.

	Criterion1	Criterion2	Criterion3	Criterion4	Criterion5
Alter-1	<[0.65, 0.70], [0.15, 0.25]>	<[0.75, 0.80], [0.15, 0.20]>	<[0.75, 0.80], [0.15, 0.20]>	<[0.65, 0.70], [0.20, 0.25]>	<[0.75, 0.80], [0.15, 0.20]>
Alter-2	<[0.70, 0.75], [0.20, 0.25]>	<[0.90, 0.95], [0.00, 0.05]>	<[0.90, 0.95], [0.00, 0.05]>	<[0.85, 0.90], [0.05, 0.10]>	<[0.65, 0.70], [0.15, 0.20]>
Alter-3	<[0.75, 0.80], [0.15, 0.20]>	<[0.85, 0.90], [0.05, 0.10]>	<[0.80, 0.85], [0.10, 0.15]>	<[0.75, 0.80], [0.10, 0.00]>	<[0.70, 0.75], [0.20, 0.25]>
Alter-4	<[0.60, 0.65], [0.30, 0.35]>	<[0.65, 0.70], [0.25, 0.30]>	<[0.70, 0.75], [0.20, 0.25]>	<[0.60, 0.65], [0.30, 0.35]>	<[0.60, 0.65], [0.25, 0.30]>

**Table A15:** The decision result given by DM_14_.

	Criterion1	Criterion2	Criterion3	Criterion4	Criterion5
Alter-1	<[0.85, 0.90], [0.05, 0.10]>	<[0.75, 0.80], [0.15, 0.20]>	<[0.75, 0.80], [0.15, 0.20]>	<[0.80, 0.85], [0.10, 0.15]>	<[0.75, 0.80], [0.05, 0.15]>
Alter-2	<[0.80, 0.85], [0.10, 0.15]>	<[0.65, 0.75], [0.15, 0.20]>	<[0.70, 0.80], [0.10, 0.15]>	<[0.75, 0.85], [0.10, 0.15]>	<[0.65, 0.70], [0.20, 0.30]>
Alter-3	<[0.90, 0.95], [0.00, 0.05]>	<[0.85, 0.90], [0.05, 0.10]>	<[0.85, 0.90], [0.05, 0.10]>	<[0.85, 0.90], [0.05, 0.10]>	<[0.80, 0.85], [0.05, 0.10]>
Alter-4	<[0.05, 0.55], [0.25, 0.35]>	<[0.45, 0.50], [0.20, 0.45]>	<[0.60, 0.65], [0.25, 0.30]>	<[0.50, 0.55], [0.10, 0.35]>	<[0.40, 0.50], [0.40, 0.50]>

**Table A16:** The decision result given by DM_15_.

	Criterion1	Criterion2	Criterion3	Criterion4	Criterion5
Alter-1	<[0.65, 0.75], [0.15, 0.20]>	<[0.60, 0.65], [0.20, 0.25]>	<[0.60, 0.70], [0.15, 0.20]>	<[0.60, 0.65], [0.10, 0.20]>	<[0.60, 0.65], [0.20, 0.30]>
Alter-2	<[0.75, 0.85], [0.10, 0.15]>	<[0.60, 0.65], [0.20, 0.25]>	<[0.65, 0.70], [0.20, 0.25]>	<[0.80, 0.85], [0.10, 0.15]>	<[0.55, 0.65], [0.25, 0.30]>
Alter-3	<[0.60, 0.65], [0.25, 0.30]>	<[0.65, 0.70], [0.20, 0.30]>	<[0.60, 0.65], [0.20, 0.35]>	<[0.55, 0.60], [0.25, 0.30]>	<[0.65, 0.75], [0.20, 0.25]>
Alter-4	<[0.55, 0.60], [0.20, 0.30]>	<[0.70, 0.75], [0.10, 0.20]>	<[0.55, 0.65], [0.10, 0.25]>	<[0.45, 0.50], [0.40, 0.45]>	<[0.70, 0.75], [0.20, 0.25]>

**Table A17:** The decision result given by DM_16_.

	Criterion1	Criterion2	Criterion3	Criterion4	Criterion5
Alter-1	<[0.65, 0.75], [0.15, 0.20]>	<[0.60, 0.65], [0.20, 0.25]>	<[0.60, 0.70], [0.15, 0.20]>	<[0.60, 0.65], [0.10, 0.20]>	<[0.60, 0.65], [0.20, 0.30]>
Alter-2	<[0.55, 0.65], [0.30, 0.35]>	<[0.55, 0.60], [0.25, 0.30]>	<[0.55, 0.70], [0.15, 0.25]>	<[0.65, 0.70], [0.10, 0.15]>	<[0.60, 0.65], [0.20, 0.30]>
Alter-3	<[0.60, 0.65], [0.25, 0.30]>	<[0.65, 0.70], [0.20, 0.30]>	<[0.60, 0.65], [0.20, 0.35]>	<[0.55, 0.60], [0.25, 0.30]>	<[0.65, 0.75], [0.20, 0.25]>
Alter-4	<[0.85, 0.90], [0.05, 0.10]>	<[0.75, 0.80], [0.10, 0.20]>	<[0.70, 0.75], [0.10, 0.15]>	<[0.65, 0.70], [0.25, 0.30]>	<[0.65, 0.70], [0.15, 0.20]>

**Table A18:** The decision result given by DM_17_.

	Criterion1	Criterion2	Criterion3	Criterion4	Criterion5
Alter-1	<[0.60, 0.70], [0.15, 0.20]>	<[0.60, 0.70], [0.20, 0.25]>	<[0.60, 0.70], [0.20, 0.25]>	<[0.75, 0.80], [0.10, 0.20]>	<[0.65, 0.75], [0.20, 0.25]>
Alter-2	<[0.50, 0.60], [0.30, 0.35]>	<[0.55, 0.65], [0.25, 0.35]>	<[0.50, 0.60], [0.25, 0.35]>	<[0.65, 0.70], [0.20, 0.25]>	<[0.65, 0.70], [0.25, 0.30]>
Alter-3	<[0.50, 0.55], [0.35, 0.40]>	<[0.55, 0.60], [0.25, 0.30]>	<[0.50, 0.60], [0.35, 0.40]>	<[0.55, 0.60], [0.30, 0.35]>	<[0.55, 0.60], [0.30, 0.35]>
Alter-4	<[0.70, 0.75], [0.20, 0.25]>	<[0.70, 0.80], [0.15, 0.20]>	<[0.75, 0.80], [0.10, 0.20]>	<[0.80, 0.85], [0.05, 0.15]>	<[0.85, 0.90], [0.05, 0.10]>

**Table A19:** The decision result given by DM_18_.

	Criterion1	Criterion2	Criterion3	Criterion4	Criterion5
Alter-1	<[0.60, 0.65], [0.25, 0.30]>	<[0.65, 0.70], [0.25, 0.25]>	<[0.60, 0.65], [0.30, 0.35]>	<[0.75, 0.80], [0.15, 0.20]>	<[0.70, 0.75], [0.20, 0.25]>
Alter-2	<[0.55, 0.60], [0.30, 0.35]>	<[0.60, 0.65], [0.30, 0.35]>	<[0.55, 0.60], [0.25, 0.35]>	<[0.65, 0.70], [0.20, 0.25]>	<[0.65, 0.70], [0.25, 0.30]>
Alter-3	<[0.50, 0.55], [0.35, 0.40]>	<[0.55, 0.60], [0.20, 0.30]>	<[0.50, 0.60], [0.30, 0.40]>	<[0.55, 0.60], [0.25, 0.35]>	<[0.55, 0.60], [0.30, 0.35]>
Alter-4	<[0.70, 0.75], [0.20, 0.25]>	<[0.70, 0.80], [0.15, 0.15]>	<[0.75, 0.80], [0.10, 0.20]>	<[0.80, 0.85], [0.05, 0.15]>	<[0.85, 0.90], [0.05, 0.10]>

**Table A20:** The decision result given by DM_19_.

	Criterion1	Criterion2	Criterion3	Criterion4	Criterion5
Alter-1	<[0.55, 0.65], [0.20, 0.30]>	<[0.60, 0.70], [0.25, 0.30]>	<[0.60, 0.65], [0.30, 0.35]>	<[0.75, 0.80], [0.15, 0.20]>	<[0.70, 0.75], [0.20, 0.25]>
Alter-2	<[0.55, 0.65], [0.25, 0.35]>	<[0.55, 0.65], [0.25, 0.35]>	<[0.55, 0.65], [0.25, 0.35]>	<[0.65, 0.70], [0.20, 0.25]>	<[0.65, 0.70], [0.25, 0.30]>
Alter-3	<[0.50, 0.60], [0.30, 0.40]>	<[0.60, 0.65], [0.20, 0.25]>	<[0.50, 0.55], [0.30, 0.40]>	<[0.55, 0.60], [0.25, 0.35]>	<[0.55, 0.60], [0.30, 0.35]>
Alter-4	<[0.65, 0.75], [0.15, 0.25]>	<[0.70, 0.75], [0.10, 0.20]>	<[0.70, 0.80], [0.10, 0.20]>	<[0.75, 0.85], [0.05, 0.15]>	<[0.80, 0.90], [0.00, 0.10]>

**Table A21:** The decision result given by DM_20_.

	Criterion1	Criterion2	Criterion3	Criterion4	Criterion5
Alter-1	<[0.60, 0.65], [0.25, 0.30]>	<[0.65, 0.70], [0.25, 0.25]>	<[0.55, 0.65], [0.30, 0.35]>	<[0.70, 0.80], [0.15, 0.20]>	<[0.65, 0.75], [0.20, 0.25]>
Alter-2	<[0.55, 0.60], [0.30, 0.35]>	<[0.60, 0.65], [0.30, 0.35]>	<[0.55, 0.60], [0.25, 0.35]>	<[0.65, 0.70], [0.20, 0.25]>	<[0.60, 0.70], [0.25, 0.30]>
Alter-3	<[0.50, 0.55], [0.35, 0.40]>	<[0.55, 0.60], [0.20, 0.30]>	<[0.60, 0.65], [0.20, 0.25]>	<[0.55, 0.65], [0.25, 0.35]>	<[0.55, 0.60], [0.25, 0.30]>
Alter-4	<[0.70, 0.75], [0.20, 0.25]>	<[0.70, 0.80], [0.15, 0.20]>	<[0.75, 0.80], [0.10, 0.20]>	<[0.80, 0.85], [0.05, 0.15]>	<[0.80, 0.85], [0.10, 0.15]>

**Table A22:** The decision result given by DM_21_.

	Criterion1	Criterion2	Criterion3	Criterion4	Criterion5
Alter-1	<[0.85, 0.90], [0.05, 0.10]>	<[0.80, 0.85], [0.10, 0.15]>	<[0.65, 0.75], [0.15, 0.25]>	<[0.85, 0.90], [0.05, 0.10]>	<[0.70, 0.75], [0.20, 0.25]>
Alter-2	<[0.65, 0.75], [0.20, 0.25]>	<[0.75, 0.80], [0.10, 0.15]>	<[0.60, 0.65], [0.30, 0.35]>	<[0.75, 0.80], [0.15, 0.20]>	<[0.65, 0.70], [0.15, 0.20]>
Alter-3	<[0.75, 0.80], [0.10, 0.15]>	<[0.85, 0.90], [0.05, 0.10]>	<[0.70, 0.80], [0.10, 0.20]>	<[0.80, 0.85], [0.10, 0.15]>	<[0.80, 0.85], [0.10, 0.15]>
Alter-4	<[0.60, 0.65], [0.20, 0.25]>	<[0.70, 0.75], [0.20, 0.25]>	<[0.55, 0.60], [0.35, 0.40]>	<[0.65, 0.70], [0.25, 0.30]>	<[0.55, 0.60], [0.20, 0.30]>

**Table A23:** The decision result given by DM_22_.

	Criterion1	Criterion2	Criterion3	Criterion4	Criterion5
Alter-1	<[0.75, 0.80], [0.15, 0.20]>	<[0.70, 0.80], [0.15, 0.20]>	<[0.75, 0.80], [0.10, 0.20]>	<[0.70, 0.75], [0.20, 0.25]>	<[0.85, 0.90], [0.05, 0.10]>
Alter-2	<[0.65, 0.70], [0.20, 0.25]>	<[0.65, 0.70], [0.20, 0.25]>	<[0.65, 0.70], [0.25, 0.30]>	<[0.65, 0.70], [0.25, 0.30]>	<[0.75, 0.80], [0.15, 0.20]>
Alter-3	<[0.80, 0.85], [0.05, 0.10]>	<[0.75, 0.80], [0.15, 0.20]>	<[0.80, 0.85], [0.05, 0.15]>	<[0.80, 0.85], [0.10, 0.15]>	<[0.80, 0.85], [0.10, 0.15]>
Alter-4	<[0.55, 0.65], [0.30, 0.35]>	<[0.60, 0.65], [0.20, 0.25]>	<[0.60, 0.65], [0.30, 0.35]>	<[0.60, 0.65], [0.30, 0.35]>	<[0.65, 0.70], [0.20, 0.30]>

**Table A24:** The decision result given by DM_23_.

	Criterion1	Criterion2	Criterion3	Criterion4	Criterion5
Alter-1	<[0.75, 0.80], [0.15, 0.20]>	<[0.85, 0.90], [0.10, 0.15]>	<[0.75, 0.80], [0.15, 0.20]>	<[0.75, 0.80], [0.15, 0.20]>	<[0.75, 0.90], [0.10, 0.10]>
Alter-2	<[0.65, 0.70], [0.25, 0.30]>	<[0.75, 0.80], [0.15, 0.20]>	<[0.80, 0.85], [0.10, 0.15]>	<[0.65, 0.70], [0.25, 0.30]>	<[0.60, 0.75], [0.15, 0.20]>
Alter-3	<[0.55, 0.65], [0.25, 0.30]>	<[0.65, 0.70], [0.25, 0.30]>	<[0.70, 0.75], [0.20, 0.25]>	<[0.50, 0.55], [0.40, 0.45]>	<[0.55, 0.70], [0.20, 0.25]>
Alter-4	<[0.85, 0.90], [0.05, 0.10]>	<[0.90, 0.95], [0.00, 0.05]>	<[0.85, 0.90], [0.05, 0.10]>	<[0.85, 0.90], [0.05, 0.10]>	<[0.65, 0.85], [0.05, 0.15]>

**Table A25:** The decision result given by DM_24_.

	Criterion1	Criterion2	Criterion3	Criterion4	Criterion5
Alter-1	<[0.65, 0.70], [0.20, 0.30]>	<[0.85, 0.90], [0.05, 0.10]>	<[0.90, 0.95], [0.00, 0.05]>	<[0.80, 0.85], [0.10, 0.15]>	<[0.95, 0.70], [0.25, 0.30]>
Alter-2	<[0.60, 0.65], [0.25, 0.35]>	<[0.60, 0.65], [0.30, 0.35]>	<[0.70, 0.75], [0.20, 0.25]>	<[0.70, 0.75], [0.20, 0.25]>	<[0.75, 0.65], [0.60, 0.35]>
Alter-3	<[0.55, 0.60], [0.20, 0.25]>	<[0.65, 0.70], [0.25, 0.30]>	<[0.65, 0.70], [0.15, 0.20]>	<[0.65, 0.70], [0.25, 0.30]>	<[0.60, 0.55], [0.35, 0.45]>
Alter-4	<[0.70, 0.75], [0.20, 0.25]>	<[0.70, 0.75], [0.05, 0.10]>	<[0.85, 0.90], [0.05, 0.10]>	<[0.75, 0.80], [0.10, 0.15]>	<[0.85, 0.80], [0.15, 0.15]>

**Table A26:** The decision result given by DM_25_.

	Criterion1	Criterion2	Criterion3	Criterion4	Criterion5
Alter-1	<[0.65, 0.75], [0.20, 0.25]>	<[0.60, 0.65], [0.25, 0.30]>	<[0.80, 0.85], [0.05, 0.10]>	<[0.80, 0.85], [0.00, 0.15]>	<[0.75, 0.80], [0.10, 0.15]>
Alter-2	<[0.60, 0.65], [0.20, 0.25]>	<[0.55, 0.60], [0.25, 0.35]>	<[0.65, 0.70], [0.25, 0.30]>	<[0.70, 0.75], [0.15, 0.25]>	<[0.65, 0.70], [0.20, 0.25]>
Alter-3	<[0.60, 0.65], [0.25, 0.30]>	<[0.55, 0.60], [0.25, 0.35]>	<[0.55, 0.60], [0.30, 0.35]>	<[0.65, 0.70], [0.20, 0.25]>	<[0.60, 0.65], [0.25, 0.35]>
Alter-4	<[0.75, 0.85], [0.10, 0.15]>	<[0.75, 0.80], [0.10, 0.20]>	<[0.85, 0.90], [0.05, 0.10]>	<[0.85, 0.90], [0.10, 0.05]>	<[0.70, 0.75], [0.20, 0.25]>

**Table A27:** The decision result given by DM_26_.

	Criterion1	Criterion2	Criterion3	Criterion4	Criterion5
Alter-1	<[0.60, 0.70], [0.20, 0.30]>	<[0.70, 0.75], [0.20, 0.25]>	<[0.70, 0.75], [0.20, 0.25]>	<[0.55, 0.60], [0.30, 0.35]>	<[0.65, 0.70], [0.25, 0.30]>
Alter-2	<[0.80, 0.85], [0.10, 0.15]>	<[0.65, 0.70], [0.20, 0.25]>	<[0.75, 0.80], [0.15, 0.20]>	<[0.85, 0.90], [0.00, 0.10]>	<[0.55, 0.60], [0.20, 0.30]>
Alter-3	<[0.60, 0.65], [0.20, 0.30]>	<[0.65, 0.70], [0.10, 0.15]>	<[0.65, 0.75], [0.20, 0.25]>	<[0.70, 0.75], [0.10, 0.15]>	<[0.75, 0.80], [0.10, 0.15]>
Alter-4	<[0.55, 0.65], [0.05, 0.15]>	<[0.60, 0.65], [0.20, 0.25]>	<[0.60, 0.65], [0.20, 0.30]>	<[0.55, 0.60], [0.20, 0.30]>	<[0.60, 0.70], [0.20, 0.25]>

**Table A28:** The decision result given by DM_27_.

	Criterion1	Criterion2	Criterion3	Criterion4	Criterion5
Alter-1	<[0.25, 0.35], [0.45, 0.55]>	<[0.55, 0.60], [0.35, 0.40]>	<[0.15, 0.30], [0.60, 0.65]>	<[0.45, 0.50], [0.40, 0.50]>	<[0.35, 0.40], [0.25, 0.35]>
Alter-2	<[0.55, 0.65], [0.10, 0.30]>	<[0.35, 0.40], [0.25, 0.30]>	<[0.40, 0.50], [0.40, 0.45]>	<[0.35, 0.50], [0.30, 0.35]>	<[0.45, 0.50], [0.20, 0.35]>
Alter-3	<[0.65, 0.75], [0.10, 0.20]>	<[0.55, 0.65], [0.20, 0.30]>	<[0.30, 0.40], [0.30, 0.40]>	<[0.50, 0.55], [0.25, 0.40]>	<[0.55, 0.65], [0.15, 0.35]>
Alter-4	<[0.45, 0.55], [0.25, 0.35]>	<[0.45, 0.50], [0.20, 0.45]>	<[0.60, 0.65], [0.25, 0.30]>	<[0.50, 0.55], [0.10, 0.35]>	<[0.40, 0.50], [0.40, 0.50]>

**Table A29:** The decision result given by DM_28_.

	Criterion1	Criterion2	Criterion3	Criterion4	Criterion5
Alter-1	<[0.70, 0.80], [0.15, 0.20]>	<[0.65, 0.70], [0.20, 0.25]>	<[0.65, 0.70], [0.25, 0.30]>	<[0.70, 0.75], [0.20, 0.25]>	<[0.85, 0.90], [0.05, 0.10]>
Alter-2	<[0.80, 0.90], [0.05, 0.10]>	<[0.70, 0.75], [0.20, 0.25]>	<[0.75, 0.80], [0.15, 0.20]>	<[0.75, 0.85], [0.10, 0.15]>	<[0.90, 0.95], [0.00, 0.05]>
Alter-3	<[0.70, 0.75], [0.20, 0.25]>	<[0.60, 0.65], [0.25, 0.30]>	<[0.60, 0.65], [0.25, 0.30]>	<[0.65, 0.70], [0.15, 0.20]>	<[0.80, 0.85], [0.10, 0.15]>
Alter-4	<[0.65, 0.70], [0.25, 0.30]>	<[0.55, 0.65], [0.30, 0.35]>	<[0.55, 0.60], [0.30, 0.35]>	<[0.60, 0.65], [0.25, 0.30]>	<[0.55, 0.65], [0.25, 0.30]>

**Table A30:** The decision result given by DM_29_.

	Criterion1	Criterion2	Criterion3	Criterion4	Criterion5
Alter-1	<[0.15, 0.30], [0.40, 0.45]>	<[0.40, 0.50], [0.30, 0.40]>	<[0.25, 0.45], [0.40, 0.50]>	<[0.30, 0.50], [0.40, 0.50]>	<[0.30, 0.40], [0.15, 0.20]>
Alter-2	<[0.40, 0.55], [0.15, 0.30]>	<[0.35, 0.45], [0.30, 0.45]>	<[0.45, 0.55], [0.40, 0.50]>	<[0.45, 0.50], [0.30, 0.45]>	<[0.40, 0.50], [0.35, 0.45]>
Alter-3	<[0.50, 0.65], [0.15, 0.35]>	<[0.45, 0.50], [0.25, 0.45]>	<[0.30, 0.40], [0.30, 0.40]>	<[0.50, 0.55], [0.10, 0.30]>	<[0.50, 0.65], [0.15, 0.30]>
Alter-4	<[0.45, 0.55], [0.35, 0.40]>	<[0.45, 0.65], [0.20, 0.30]>	<[0.50, 0.70], [0.20, 0.30]>	<[0.40, 0.50], [0.10, 0.35]>	<[0.45, 0.55], [0.45, 0.50]>

**Table A31:** The decision result given by DM_30_.

	Criterion1	Criterion2	Criterion3	Criterion4	Criterion5
Alter-1	<[0.60, 0.65], [0.20, 0.30]>	<[0.75, 0.80], [0.15, 0.20]>	<[0.70, 0.75], [0.20, 0.25]>	<[0.55, 0.60], [0.30, 0.35]>	<[0.65, 0.70], [0.25, 0.30]>
Alter-2	<[0.80, 0.85], [0.05, 0.10]>	<[0.65, 0.70], [0.15, 0.20]>	<[0.75, 0.80], [0.15, 0.20]>	<[0.80, 0.85], [0.05, 0.10]>	<[0.65, 0.70], [0.20, 0.30]>
Alter-3	<[0.65, 0.70], [0.20, 0.25]>	<[0.60, 0.65], [0.10, 0.15]>	<[0.65, 0.70], [0.25, 0.30]>	<[0.70, 0.75], [0.10, 0.15]>	<[0.75, 0.85], [0.10, 0.15]>
Alter-4	<[0.55, 0.60], [0.25, 0.30]>	<[0.60, 0.65], [0.20, 0.25]>	<[0.60, 0.70], [0.25, 0.30]>	<[0.60, 0.65], [0.15, 0.20]>	<[0.65, 0.70], [0.20, 0.25]>

**Table A32:** The decision result given by DM_31_.

	Criterion1	Criterion2	Criterion3	Criterion4	Criterion5
Alter-1	<[0.70, 0.75], [0.15, 0.20]>	<[0.60, 0.65], [0.25, 0.30]>	<[0.60, 0.65], [0.20, 0.25]>	<[0.75, 0.85], [0.05, 0.15]>	<[0.85, 0.90], [0.10, 0.10]>
Alter-2	<[0.80, 0.85], [0.05, 0.10]>	<[0.55, 0.60], [0.35, 0.40]>	<[0.70, 0.75], [0.20, 0.25]>	<[0.85, 0.90], [0.05, 0.10]>	<[0.80, 0.85], [0.05, 0.15]>
Alter-3	<[0.65, 0.70], [0.20, 0.25]>	<[0.65, 0.70], [0.25, 0.30]>	<[0.65, 0.70], [0.15, 0.20]>	<[0.70, 0.75], [0.20, 0.25]>	<[0.70, 0.75], [0.15, 0.20]>
Alter-4	<[0.60, 0.65], [0.30, 0.35]>	<[0.50, 0.55], [0.40, 0.45]>	<[0.55, 0.60], [0.25, 0.30]>	<[0.65, 0.70], [0.15, 0.20]>	<[0.65, 0.70], [0.20, 0.25]>

**Table A33:** The decision result given by DM_32_.

	Criterion1	Criterion2	Criterion3	Criterion4	Criterion5
Alter-1	<[0.80, 0.90], [0.05, 0.10]>	<[0.70, 0.80], [0.15, 0.20]>	<[0.75, 0.85], [0.10, 0.15]>	<[0.75, 0.85], [0.10, 0.15]>	<[0.70, 0.80], [0.05, 0.15]>
Alter-2	<[0.85, 0.95], [0.00, 0.05]>	<[0.80, 0.90], [0.05, 0.10]>	<[0.85, 0.90], [0.05, 0.10]>	<[0.80, 0.90], [0.05, 0.10]>	<[0.80, 0.85], [0.10, 0.15]>
Alter-3	<[0.80, 0.85], [0.10, 0.15]>	<[0.70, 0.75], [0.10, 0.15]>	<[0.70, 0.75], [0.15, 0.20]>	<[0.75, 0.80], [0.10, 0.20]>	<[0.65, 0.70], [0.20, 0.30]>
Alter-4	<[0.75, 0.85], [0.10, 0.15]>	<[0.60, 0.70], [0.20, 0.25]>	<[0.65, 0.70], [0.20, 0.25]>	<[0.65, 0.70], [0.25, 0.30]>	<[0.60, 0.65], [0.20, 0.35]>

**Table A34:** The decision result given by DM_33_.

	Criterion1	Criterion2	Criterion3	Criterion4	Criterion5
Alter-1	<[0.60, 0.65], [0.20, 0.25]>	<[0.60, 0.70], [0.25, 0.25]>	<[0.60, 0.65], [0.30, 0.35]>	<[0.75, 0.80], [0.15, 0.20]>	<[0.70, 0.75], [0.20, 0.25]>
Alter-2	<[0.70, 0.75], [0.20, 0.25]>	<[0.75, 0.80], [0.15, 0.20]>	<[0.75, 0.80], [0.10, 0.15]>	<[0.80, 0.90], [0.05, 0.10]>	<[0.85, 0.90], [0.05, 0.10]>
Alter-3	<[0.60, 0.65], [0.25, 0.30]>	<[0.60, 0.65], [0.30, 0.35]>	<[0.55, 0.60], [0.25, 0.35]>	<[0.65, 0.70], [0.20, 0.25]>	<[0.70, 0.75], [0.20, 0.25]>
Alter-4	<[0.50, 0.55], [0.40, 0.45]>	<[0.55, 0.65], [0.25, 0.30]>	<[0.50, 0.55], [0.40, 0.45]>	<[0.55, 0.60], [0.30, 0.35]>	<[0.60, 0.65], [0.25, 0.30]>

**Table A35:** The decision result given by DM_34_.

	Criterion1	Criterion2	Criterion3	Criterion4	Criterion5
Alter-1	<[0.75, 0.80], [0.10, 0.15]>	<[0.65, 0.70], [0.20, 0.25]>	<[0.65, 0.70], [0.25, 0.30]>	<[0.70, 0.75], [0.20, 0.25]>	<[0.85, 0.90], [0.05, 0.10]>
Alter-2	<[0.70, 0.80], [0.15, 0.20]>	<[0.60, 0.65], [0.20, 0.30]>	<[0.60, 0.65], [0.30, 0.35]>	<[0.65, 0.70], [0.15, 0.25]>	<[0.75, 0.80], [0.15, 0.20]>
Alter-3	<[0.85, 0.90], [0.05, 0.10]>	<[0.70, 0.75], [0.15, 0.20]>	<[0.70, 0.80], [0.15, 0.20]>	<[0.75, 0.80], [0.10, 0.15]>	<[0.90, 0.95], [0.00, 0.05]>
Alter-4	<[0.65, 0.75], [0.20, 0.25]>	<[0.55, 0.60], [0.30, 0.35]>	<[0.55, 0.60], [0.35, 0.40]>	<[0.60, 0.65], [0.20, 0.30]>	<[0.60, 0.65], [0.25, 0.30]>

**Table A36:** The decision result given by DM_35_.

	Criterion1	Criterion2	Criterion3	Criterion4	Criterion5
Alter-1	<[0.60, 0.65], [0.25, 0.30]>	<[0.65, 0.70], [0.25, 0.25]>	<[0.60, 0.65], [0.30, 0.35]>	<[0.75, 0.80], [0.15, 0.20]>	<[0.70, 0.75], [0.20, 0.25]>
Alter-2	<[0.55, 0.60], [0.30, 0.35]>	<[0.60, 0.65], [0.30, 0.35]>	<[0.55, 0.60], [0.25, 0.35]>	<[0.65, 0.70], [0.20, 0.25]>	<[0.65, 0.70], [0.25, 0.30]>
Alter-3	<[0.50, 0.55], [0.35, 0.40]>	<[0.55, 0.60], [0.20, 0.30]>	<[0.50, 0.60], [0.30, 0.40]>	<[0.55, 0.60], [0.25, 0.35]>	<[0.55, 0.60], [0.30, 0.35]>
Alter-4	<[0.70, 0.75], [0.20, 0.25]>	<[0.70, 0.80], [0.15, 0.20]>	<[0.75, 0.80], [0.10, 0.20]>	<[0.80, 0.85], [0.05, 0.15]>	<[0.85, 0.90], [0.05, 0.10]>

**Table A37:** The decision result given by DM_36_.

	Criterion1	Criterion2	Criterion3	Criterion4	Criterion5
Alter-1	<[0.45, 0.55], [0.20, 0.37]>	<[0.43, 0.56], [0.21, 0.38]>	<[0.45, 0.58], [0.20, 0.40]>	<[0.40, 0.57], [0.21, 0.42]>	<[0.46, 0.59], [0.21, 0.38]>
Alter-2	<[0.60, 0.68], [0.18, 0.30]>	<[0.56, 0.66], [0.20, 0.31]>	<[0.55, 0.65], [0.18, 0.33]>	<[0.50, 0.66], [0.19, 0.32]>	<[0.50, 0.62], [0.19, 0.34]>
Alter-3	<[0.55, 0.63], [0.23, 0.32]>	<[0.50, 0.62], [0.20, 0.33]>	<[0.48, 0.60], [0.17, 0.34]>	<[0.42, 0.61], [0.18, 0.33]>	<[0.47, 0.63], [0.18, 0.35]>
Alter-4	<[0.50, 0.61], [0.21, 0.36]>	<[0.51, 0.60], [0.23, 0.33]>	<[0.50, 0.61], [0.20, 0.35]>	<[0.30, 0.60], [0.21, 0.36]>	<[0.51, 0.60], [0.19, 0.36]>

**Table A38:** The decision result given by DM_37_.

	Criterion1	Criterion2	Criterion3	Criterion4	Criterion5
Alter-1	<[0.46, 0.56], [0.22, 0.36]>	<[0.42, 0.50], [0.22, 0.38]>	<[0.45, 0.58], [0.20, 0.40]>	<[0.38, 0.56], [0.20, 0.37]>	<[0.46, 0.57], [0.20, 0.37]>
Alter-2	<[0.61, 0.67], [0.17, 0.31]>	<[0.55, 0.62], [0.18, 0.28]>	<[0.45, 0.60], [0.17, 0.30]>	<[0.42, 0.62], [0.21, 0.31]>	<[0.51, 0.61], [0.21, 0.35]>
Alter-3	<[0.51, 0.64], [0.22, 0.32]>	<[0.49, 0.53], [0.19, 0.32]>	<[0.50, 0.61], [0.16, 0.32]>	<[0.41, 0.55], [0.19, 0.32]>	<[0.48, 0.62], [0.20, 0.33]>
Alter-4	<[0.51, 0.62], [0.23, 0.33]>	<[0.50, 0.60], [0.22, 0.33]>	<[0.46, 0.55], [0.21, 0.34]>	<[0.40, 0.56], [0.18, 0.30]>	<[0.41, 0.55], [0.17, 0.37]>

**Table A39:** The decision result given by DM_38_.

	Criterion1	Criterion2	Criterion3	Criterion4	Criterion5
Alter-1	<[0.35, 0.56], [0.21, 0.34]>	<[0.55, 0.58], [0.20, 0.36]>	<[0.48, 0.53], [0.22, 0.38]>	<[0.41, 0.55], [0.22, 0.41]>	<[0.50, 0.57], [0.21, 0.36]>
Alter-2	<[0.61, 0.67], [0.19, 0.31]>	<[0.46, 0.54], [0.18, 0.33]>	<[0.50, 0.62], [0.19, 0.31]>	<[0.51, 0.60], [0.21, 0.30]>	<[0.48, 0.62], [0.16, 0.37]>
Alter-3	<[0.52, 0.62], [0.20, 0.35]>	<[0.43, 0.56], [0.19, 0.32]>	<[0.43, 0.57], [0.20, 0.35]>	<[0.41, 0.63], [0.17, 0.34]>	<[0.48, 0.61], [0.17, 0.38]>
Alter-4	<[0.45, 0.56], [0.16, 0.38]>	<[0.42, 0.61], [0.21, 0.37]>	<[0.52, 0.60], [0.22, 0.34]>	<[0.31, 0.61], [0.19, 0.35]>	<[0.50, 0.60], [0.18, 0.37]>

**Table A40:** The decision result given by DM_39_.

	Criterion1	Criterion2	Criterion3	Criterion4	Criterion5
Alter-1	<[0.34, 0.53], [0.21, 0.35]>	<[0.40, 0.53], [0.20, 0.33]>	<[0.51, 0.56], [0.19, 0.34]>	<[0.41, 0.56], [0.22, 0.41]>	<[0.42, 0.58], [0.20, 0.34]>
Alter-2	<[0.56, 0.60], [0.17, 0.30]>	<[0.51, 0.56], [0.18, 0.28]>	<[0.55, 0.60], [0.16, 0.36]>	<[0.45, 0.58], [0.18, 0.39]>	<[0.45, 0.59], [0.17, 0.36]>
Alter-3	<[0.50, 0.56], [0.20, 0.33]>	<[0.53, 0.60], [0.19, 0.35]>	<[0.44, 0.61], [0.18, 0.32]>	<[0.40, 0.55], [0.20, 0.35]>	<[0.45, 0.60], [0.20, 0.33]>
Alter-4	<[0.51, 0.62], [0.22, 0.37]>	<[0.50, 0.56], [0.22, 0.32]>	<[0.45, 0.55], [0.21, 0.33]>	<[0.31, 0.61], [0.19, 0.36]>	<[0.50, 0.56], [0.20, 0.37]>

**Table A41:** The decision result given by DM_40_.

	Criterion1	Criterion2	Criterion3	Criterion4	Criterion5
Alter-1	<[0.48, 0.53], [0.21, 0.36]>	<[0.40, 0.55], [0.20, 0.39]>	<[0.43, 0.53], [0.32, 0.41]>	<[0.41, 0.53], [0.22, 0.43]>	<[0.43, 0.58], [0.20, 0.40]>
Alter-2	<[0.49, 0.65], [0.20, 0.31]>	<[0.53, 0.62], [0.19, 0.35]>	<[0.50, 0.60], [0.22, 0.35]>	<[0.51, 0.61], [0.23, 0.35]>	<[0.45, 0.60], [0.21, 0.37]>
Alter-3	<[0.50, 0.60], [0.19, 0.30]>	<[0.51, 0.60], [0.22, 0.30]>	<[0.49, 0.61], [0.26, 0.32]>	<[0.40, 0.56], [0.21, 0.37]>	<[0.43, 0.56], [0.19, 0.38]>
Alter-4	<[0.45, 0.57], [0.18, 0.33]>	<[0.47, 0.56], [0.27, 0.31]>	<[0.42, 0.48], [0.21, 0.34]>	<[0.34, 0.57], [0.19, 0.32]>	<[0.48, 0.58], [0.17, 0.39]>
